# Turning Molecular Springs into Nano-Shock Absorbers: The Effect of Macroscopic Morphology and Crystal Size on the Dynamic Hysteresis of Water Intrusion–Extrusion into-from Hydrophobic Nanopores

**DOI:** 10.1021/acsami.2c04314

**Published:** 2022-06-03

**Authors:** Paweł Zajdel, David G. Madden, Robin Babu, Marco Tortora, Diego Mirani, Nikolay Nikolaevich Tsyrin, Luis Bartolomé, Eder Amayuelas, David Fairen-Jimenez, Alexander Rowland Lowe, Mirosław Chorążewski, Juscelino B. Leao, Craig M. Brown, Markus Bleuel, Victor Stoudenets, Carlo Massimo Casciola, María Echeverría, Francisco Bonilla, Giulia Grancini, Simone Meloni, Yaroslav Grosu

**Affiliations:** † Institute of Physics, University of Silesia in Katowice, 75 Pulku Piechoty 1, 41-500 Chorzow, Poland; ‡ The Adsorption & Advanced Materials Laboratory (A^2^ML), Department of Chemical Engineering & Biotechnology, University of Cambridge, Philippa Fawcett Drive, Cambridge CB3 0AS, U.K.; § Dipartimento di Ingegneria Meccanica e Aerospaziale, Sapienza Università di Roma, via Eudossiana 18, 00184 Rome, Italy; ∥ Department of Chemistry & INSTM University of Pavia, Via Taramelli 14, Pavia I-27100, Italy; ⊥ Laboratory of Thermomolecular Energetics, National Technical University of Ukraine “Igor Sikorsky Kyiv Polytechnic Institute”, Pr. Peremogy 37, 03056 Kyiv, Ukraine; # Centre for Cooperative Research on Alternative Energies (CIC energiGUNE), Basque Research and Technology Alliance (BRTA), Albert Einstein 48, 01510 Vitoria-Gasteiz, Spain; ¶ Institute of Chemistry, University of Silesia in Katowice, Szkolna 9, 40-006 Katowice, Poland; ∇ NIST Center for Neutron Research, National Institute of Standards and Technology, Gaithersburg, Maryland 20899, United States; ○ Chemical and Biochemical Department, University of Delaware, Newark, Delaware 19716, United States; ⧫ Department of Materials Science and Engineering, University of Maryland, College Park, Maryland 20742-2115, United States; †† Dipartimento di Scienze Chimiche e Farmaceutiche (DipSCF), Università degli Studi di Ferrara (Unife), Via Luigi Borsari 46, I-44121 Ferrara, Italy

**Keywords:** intrusion−extrusion, mechanical energy conversion, metal−organic framework, nanoporous materials

## Abstract

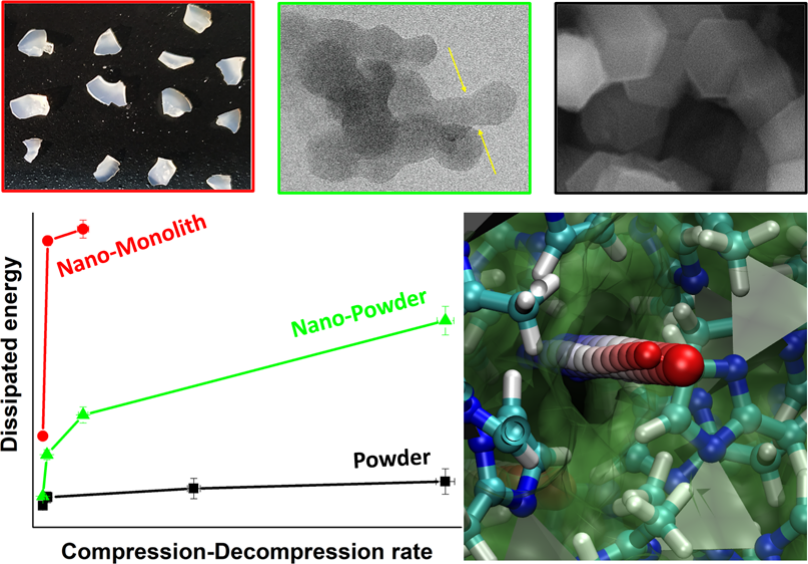

Controlling the pressure at which liquids intrude (wet) and extrude (dry) a nanopore is of paramount importance for a broad range of applications, such as energy conversion, catalysis, chromatography, separation, ionic channels, and many more. To tune these characteristics, one typically acts on the chemical nature of the system or pore size. In this work, we propose an alternative route for controlling both intrusion and extrusion pressures *via* proper arrangement of the grains of the nanoporous material. To prove the concept, dynamic intrusion–extrusion cycles for powdered and monolithic ZIF-8 metal–organic framework were conducted by means of water porosimetry and *in operando* neutron scattering. We report a drastic increase in intrusion–extrusion dynamic hysteresis when going from a fine powder to a dense monolith configuration, transforming an intermediate performance of the ZIF-8 + water system (poor molecular spring) into a desirable shock-absorber with more than 1 order of magnitude enhancement of dissipated energy per cycle. The obtained results are supported by MD simulations and pave the way for an alternative methodology of tuning intrusion–extrusion pressure using a macroscopic arrangement of nanoporous material.

## 1 Introduction

**Scheme 1:**
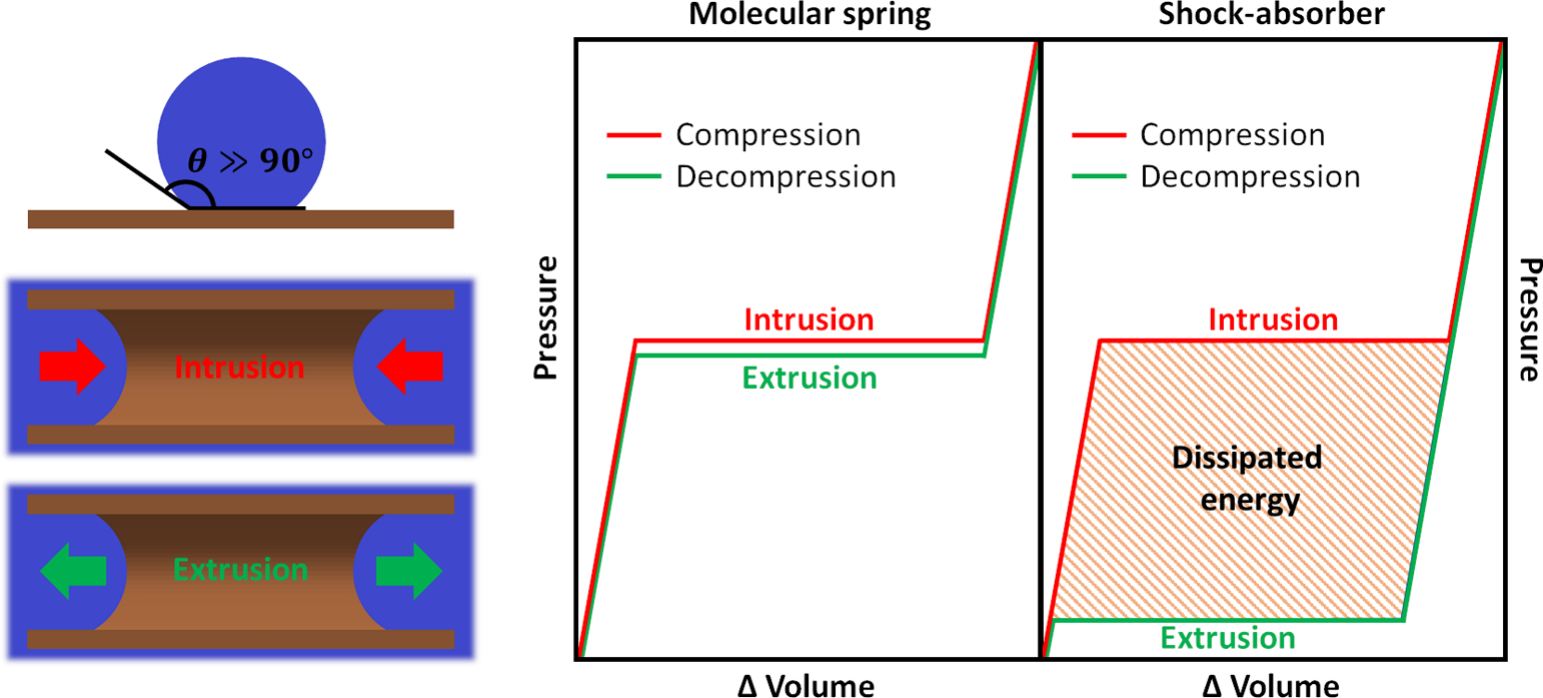
(top left): A Drop of a Non-wetting Liquid on a Lyophobic Material; (bottom left): Cross-Sectional View of a Non-Wetting Liquid as It Intrudes (Wets)/Extrudes (Dries) a Lyophobic Pore; (right): *PV*-Isotherm of an Intrusion–Extrusion Cycle with Low (Molecular Spring) and High (Shock-Absorber) Hysteresis Loops

The process of wetting-drying in nanopores is relevant to a very broad range of applications from catalysis, chromatography, and separation to ionic channels and energy conversion.^1−4^ In particular, the process of solid–liquid interface development-reduction has been widely explored for energy storage, conversion, and dissipation applications^5−9^ in view of compactness^10−12^ and an associated rapid charging–discharging cycle.^8,13,14^ The process of forced intrusion—spontaneous extrusion of a non-wetting liquid into-from a lyophobic nanopore—constitutes a charge–discharge cycle, where mechanical (work of intrusion–extrusion), thermal (heat of solid–liquid interface development-reduction), and electrical (solid–liquid triboelectrification) energies manifest themselves simultaneously.^15,16^ The operational cycle of heterogenous lyophobic systems (HLSs) is depicted in Scheme 1. Due to a non-wetting condition (Scheme 1, top left), under ambient pressure, lyophobic pores tend to stay dry and empty. However, at certain pressures, intrusion can be induced (Scheme 1, bottom left). The intrusion is associated with a plateau in the pressure-volume diagram (Scheme 1, right) and a corresponding accumulation of mechanical energy (work of compression). For the majority of HLSs, intrusion is an endothermic process,^9,15−18^ meaning that the system simultaneously accumulates thermal energy in the form of heat from the solid–liquid interface development as a non-wetting liquid spreads over a lyophobic pore (Scheme 1, bottom left). Recently, it has been demonstrated that intrusion is also associated with pronounced solid–liquid triboelectrification,^15,16^ which means that also electrical energy is generated during this process. Energetically a lyophobic pore is an unfavorable environment for a non-wetting liquid; therefore, upon decompression, the spontaneous extrusion (drying) of the pore occurs (Scheme 1, bottom left). If the extrusion pressure is similar to the intrusion pressure, the system behaves similar to a spring. Considering that breaking-forming of intermolecular bonds is at the heart of the charging–discharging process, these systems have been termed as Molecular Springs^19−21^—Scheme 1, right. These systems can be used for energy storage. Conversely, if the extrusion pressure is considerably lower than the intrusion pressure, the system behaves as a shock-absorber^8,13^ and can be used for energy dissipation applications—Scheme 1, right.

Typical examples of shock-absorber HLSs include mesoporous grafted silica gels + water/aqueous solutions^22−27^ and recently extensions were made to metal–organic framework (MOF) + water/aqueous solution systems.^28^ MOFs, due to their unique mechanical properties,^29,30^ allow additional tuning of the intrusion–extrusion process and novel applications.^16,31^ Alternatives to water/aqueous systems have also been studied using ionic liquid,^32^ ferromagnetic fluids^33^ or glycerin, and glycerol.^34,35^ These systems demonstrated good reproducibility and durability^8,14^ and were rather quickly used as a basis to construct novel shock-absorbers and bumpers.^13,36,37^ On the other hand, examples for molecular spring behavior have only been reported for the intrusion of water/aqueous solutions into a handful of microporous materials such as zeolites^38−40^ and MOFs.^21^

From these examples, it is evident that a hysteresis loop in *PV*-diagrams for HLS (Scheme 1) defines its technological applicability: storage by molecular springs or dissipation by shock-absorbers. With this in mind, several strategies have been utilized to tune the intrusion–extrusion hysteresis such as salt concentration in a non-wetting liquid,^28,41−43^ viscosity,^32^ topology of a lyophobic porous material,^44^ and porous materials’ flexibility.^16^ Each of these strategies obviously require the careful selection of a proper porous material and/or non-wetting liquids.

In this work, we demonstrate, for the first time, that a macroscopic grain arrangement and a crystal size of a nanoporous material can be used to drastically affect the dynamic hysteresis of the intrusion–extrusion process. To demonstrate the concept, we have combined water intrusion–extrusion with *in operando* neutron diffraction experiments for one of a benchmark metal–organic frameworks (MOFs), highly hydrophobicity zeolitic imidazolate framework—8 (ZIF-8), prepared in three configurations—a fine powder of macroscopic crystals (_powder_ZIF-8), a fine powder of nanoscopic crystals (_powder_nano_ZIF-8), and a dense monolith made of nanoscopic crystals (_mono_nano_ZIF-8).^45^ The obtained results demonstrate that by densely packing grains of ZIF-8, one can effectively transform a molecular spring into a nano-shock-absorber, enhancing the amount of dissipated energy per cycle by more than 1 order of magnitude. This opens a new route for tuning heterogeneous lyophobic systems for energy storage/dissipation applications solely by varying the macroscopic grain arrangement while maintaining the same porous material and a non-wetting liquid.

## 2 Materials and Methods

### 2.1 Materials

Three porous MOF materials were used in this work: powdered ZIF-8 (_powder_ZIF-8), which was purchased from Sigma-Aldrich as Basolite Z1200, CAS# 59061-53-9, monolithic ZIF-8 (_mono_nano_ZIF-8), synthesized using the previously reported method^46^ and powder of nanoscopic crystals (_powder_nano_ZIF-8). In a typical _mono_ZIF-8 synthesis, solutions containing 2-methylimidazole (20 mL, 0.395 M) and Zn(NO_3_)·6H_2_O (20 mL, 0.049 M) in ethanol were mixed and stirred for 15 min at room temperature. The mixture is then transferred to a 50 mL Falcon tube and centrifuged ( Hz) for 10 min before decanting the excess ethanol and replacing it with 10 mL of fresh ethanol and centrifuged again. This process was repeated three times. After centrifuging, a white solid was collected and dried at room temperature overnight to form glassy-looking, transparent _mono_ZIF-8. Fully activated MOF materials were obtained by heating to 120 °C under vacuum for 12 h. The synthesis of _powder_nano_ZIF-8 was performed as follows. Zn(NO_3_)_2_·6H_2_O, 2-methylimidazole, and methanol were bought from Sigma-Aldrich and used as received. To prepare ZIF-8 nanoparticles, two methanolic precursor solutions of the metal and the ligand are prepared in two different Erlenmeyer flasks: solution A, Zn(NO_3_)_2_·6H_2_O 1.467 g in 100 mL of methanol (0.049 M); solution B, 2-methylimidazole 3.245 g in 100 mL of methanol (0.395 M). The two solutions are separately mixed until complete dissolution of the components. Solution A is then rapidly poured into solution B under stirring. The obtained solution is kept under vigorous stirring for 5 min, a cloudy product is observed to be formed. The cloudy suspension is quickly poured in four different 50 mL Falcon vials and centrifuged at 150 Hz for 30 min. The supernatant solution is disposed and the product in a pellet form is washed two times with fresh methanol (60 and 30 mL) and centrifuged each time at 150 Hz for 60 min. After the last centrifugation, the pellet product is left to dry at room temperature and finely crushed with a mortar to get a homogeneous white powder.

### 2.2 Methods

#### 2.2.1 Equipment for Dynamic *PV*-Isotherms

The *PV*-isotherms were measured at a temperature of 295 K using two different experimental setups. For the experiments which were conducted using the pressurization rate, within the range of 0.1–1 MPa min^–1^, a *PVT*-scanning transitiometer,^47,48^ constructed by BGRTech, was used. The liquid suspension samples were prepared by first weighing the solid into a stainless-steel capsule and then submerging it into water. Negative relative pressure was applied to evacuate the gas from the capsule creating the suspension. The steel vessel was then placed into a calorimetric vessel where the sample was subject to a minimum of three pressurization cycles to ensure repeatability. For the experiments involving the pressurization rate of up to 1000 MPa min^–1^, *PVT*-stand developed at the National Technical University of Ukraine “Igor Sikorsky Kyiv Polytechnic Institute” was used.^22,49^ Because of different pressure-transmitting fluids and their different quantities in the hydraulic line for each of the instruments, the *PV*-isotherms were adjusted with the following the equation in order to eliminate the effect of pressure-transmitting fluid compressibility
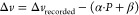


The values of  (cm^3^ g^–1^) are the original *PV*-isotherm data, where α and β are fitting constants adjusted to reach a similar volume variation upon compression (similar compressibility) below the intrusion pressure. Scaling with this method preserves the apparent pore volume (intrusion/extrusion volume) of each *PV*-isotherm and permits the direct comparison of individual *PV*-isotherms regardless of equipment used.

To avoid any relaxation effects of the ZIF-8 framework,^50^ sufficient time between cycles was provided. The time required for such relaxation was estimated from our neutrons scattering experiments (see Section 3.3 below). The *PV*-isotherms for monolith samples were recorded for multiple grains all of which are demonstrated together with their size distribution below.

#### 2.2.2 In Situ Small-Angle Neutron Scattering

In situ small-angle neutron scattering (SANS) was carried out at the National Institute of Standards and Technology (NIST) Center for Neutron Research (NCNR, Gaithersburg, USA) using the 45 m vSANS instrument. A complete description of the instrument is located at the NCNR website (https://www.nist.gov/ncnr). A neutron wavelength λ = 4.75 Å was selected using a double reflection pyrolytic graphite monochromator with a wavelength spread Δλ/λ of 0.95%. ZIF-8 was first outgassed, ex situ using a turbomolecular pump at 95 °C down to 1.2 × 10^–6^ hPa and transferred into a dry He glovebox. 197.8 mg of the sample was loaded into a SANS block pressure cell equipped with 2 mm × 2.07 mm Ti windows and 2 mm × 3.0 mm SiO_2_ spacers to decrease scattering from H_2_O (https://www.nist.gov/ncnr/sample-environment/equipment/gas-loading/situ-gas-adsorption). An additional 496.3 mg of ZIF-8 was used to fill a cylindrical steel pressure cell (https://www.nist.gov/ncnr/sample-environment/equipment/gas-loading/situ-gas-adsorption). Before each experiment, the cell was additionally outgassed of He gas at RT using a turbomolecular pump. A D_2_O/H_2_O 2:1 mixture was prepared from Cambridge Isotope Labs D_2_O (99.9%, lot M5421) and deionized water (resistivity: 15–20 MΩ, organic content: 20–50 ppm, particulate matter: <0.2 μm). The mixture was then transferred into an evacuated ISCO 100HLX syringe pump which applied and dynamically kept constant pressure (within 0.05 MPa) within each cell. A variable offset between the applied pressure and the sensor was less than 0.2 MPa. The temperature was maintained using a glycol-water bath (NESLAB RTE7) within 0.1 K for ZIF-8.

The collected data set for each pressure includes one scattering run of 900 s for ZIF-8 with a respective transmission data set of 100 s. The vSANS data were reduced using NCNR SANS macros^51^ and analyzed and visualized using DAVE.^52^

#### 2.2.3 Scanning Electron Microscopy and Transmission Electron Microscopy

A Thermo Fisher Quanta 200 FEG high-resolution scanning electron microscope was used in high-vacuum mode with electron beam energies of 10, 20, and 30 kV with a backscattered electron detector and Everhart–Thornley detector to image ZIF-8 samples. To avoid drift during image acquisition, sample was gold-coated by means of a Sputter/Carbon Coater from SPI Supplies. The operating plasma current was set at 17.1 mA in Edge Mode for 1 min.

Transmission electron microscopy (TEM) measurements were performed on the Tecnai G2 F20 Super Twin (S-Twin), a high-resolution TEM/STEM from the Thermo Fisher company with a field emission gun (FEG) and acceleration voltage of 200 kV. Samples were dispersed directly on TEM Cu grids with C mesh.

#### 2.2.4 X-ray Diffraction

A Bruker D8 Discover X-ray diffractometer was used with a LYNXEYE-XE detector using Cu Kα_1_ radiation (λ = 1.5418 Å) and Bragg–Brentano θ/2θ geometry. The data collection was carried out at room temperature, between 10 and 80° with a step of 0.02° and a dwell time of 1.03 s per step.

#### 2.2.5 77 K N_2_ Adsorption/Desorption Isotherms

N_2_ adsorption isotherms were undertaken at 77 K using a Micromeritics 3Flex and Micromeritics ASAP 2460 instruments. The temperature was controlled using a L-N_2_ bath. Brunauer, Emmett, and Teller (BET) areas were calculated using the BETSI^53^ and the Rouquerol criteria.^54^

#### 2.2.6 Mercury Porosimetry

Considering that mercury at ambient pressure does not penetrate any porosity of ZIF-8 (nor cage of ZIF-8 nor porosity formed by interparticle space), Archimedes’ method allows measuring the total volume of the sample. Next, bulk densities of the samples can be calculated by dividing the mass of the sample by the total volume. The bulk density of ZIF-8 was measured using Auto Pore IV 9500 mercury porosimeter (Micromeritics Instrument Corporation, USA) following the method described in our previous work.^46^

#### 2.2.7 Thermogravimetric Analysis

Thermogravimetry analysis was performed using the NETZSCH STA 449 F3 Jupiter thermal analyzer under a constant argon flow of 60 mL/min in the temperature range of 25–850 °C with a heating rate of 10 °C/min.

#### 2.2.8 Climatic Chamber

The hydrophilic of the samples were examined by combining exposure to 90% humidity in the Binder Model MKFT 115 climatic chamber, followed by thermogravimetric analysis. Around 30 mg of each sample was placed in a vial inside the climatic chamber at 30 °C at 90% humidity for 24 h. After that, samples were kept in hermetically closed vials and then measured by thermogravimetric analysis up to 1000 °C as described in a previous section.

#### 2.2.9 Molecular Dynamic Simulations

Simulations of the ZIF-8 grain boundary (GB) were performed within the density functional theory (DFT)^55,56^ using the Thonhauser et al. exchange and correlation (xc) functional^57−60^ implementing van der Waals interactions in DFT. This xc functional has been validated on metal–organic frameworks,^57^ the same class of materials considered in this work. The interaction between valence electrons and nuclei plus core electrons is treated using Rappe–Karin–Rabe–Kaxiras–Joannopoulos soft pseudopotentials,^61^ which allowed us to use a relatively small 40 Ry cutoff on the maximum kinetic energy of the plane waves used to expand Kohn–Sham orbitals. Given the large size of the sample, the Brillouin zone has been sampled by the single Γ point (*vide infra*). For the structure of the GBs, two (110) slab crystallites with armchair termination,^62^ containing as many as 1200 atoms each, were faced to each other. At each value of the (nominal) distance, which was controlled by keeping fixed selected atoms in the center of each ZIF-8 slab, the structure is left free to relax to the conditional (fixed nominal distance) equilibrium structure. The intrusion and percolation of a single water molecule was investigated by applying the string method,^63,64^ which allows identification of the most likely path to go from an initial to a final configuration, and the energetics of the process. A detailed description of the string method is provided in the Supporting Information.

The effect of GB-induction by constraining ZIF-8 expansion upon water intrusion has been investigated through the restrained molecular dynamics (RMD) approach,^65,66^ which allowed to compute the free energy of the system as a function of the number of water molecules in the computational MOF sample. RMD is described in detail in the Supporting Information. RMD simulations are based on classical force fields, an approach that has already been successfully applied to study intrusion–extrusion in simpler and more complex porous solids.^67−72^ Concerning the force fields, for ZIF-8, we used the force model proposed by Zheng et al.,^73^ while for water, we used the TIP5P model.^74^ Following previous works of some of us and other authors, cross interactions between water and ZIF-8 resulted from electrostatics and Lennard-Jones forces whose parameters were obtained from the standard Lorentz–Berthelot combination rules. In the case of the flexible sample, molecular dynamics is performed at a constant pressure, with the simulation box that is allowed to change along all lattice directions. On the contrary, in the case of the rigid framework, the simulation box changes only in the direction orthogonal to the slab ZIF-8 sample, preventing any overall expansion and compression of the computational crystallite in the other two directions. In both the flexible and rigid cases, atoms evolve at a constant temperature, that is, no further constraint is imposed on the atoms apart from the fixed simulation box in the slab plane for the rigid case.

## 3 Results and Discussion

### 3.1 Material Characterization

**Figure 1:**
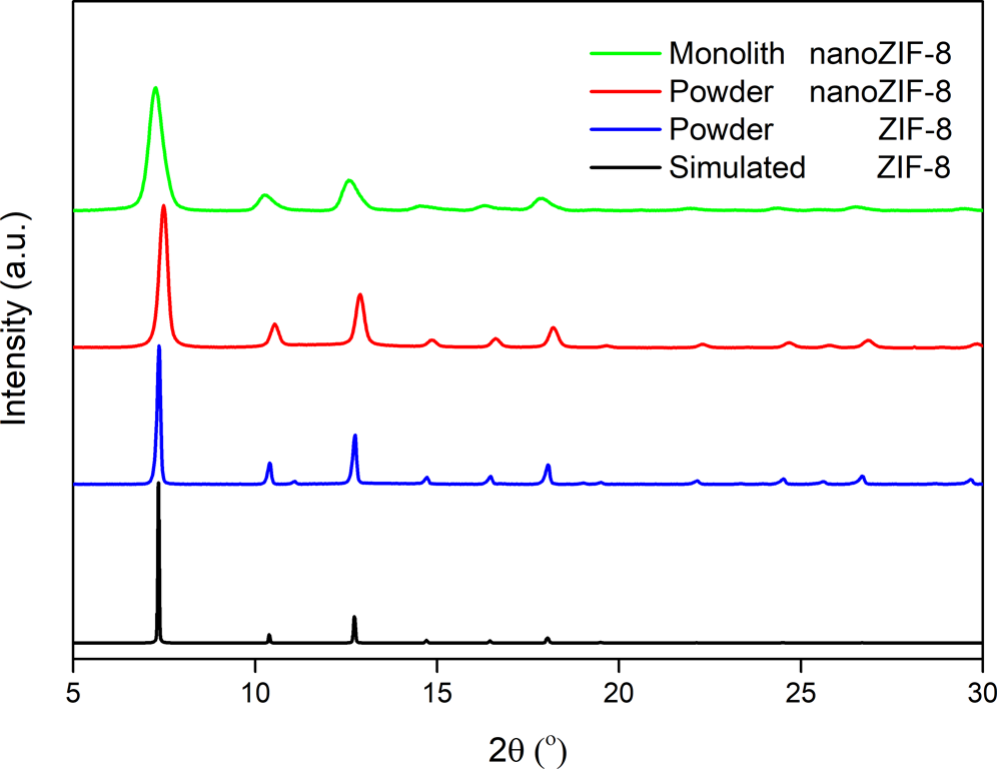
Cu Kα XRD patterns of _powder_ZIF-8, _powder_nano_ZIF-8, and _mono_nano_ZIF-8 compared to the simulated pattern of ZIF-8.

**Figure 2:**
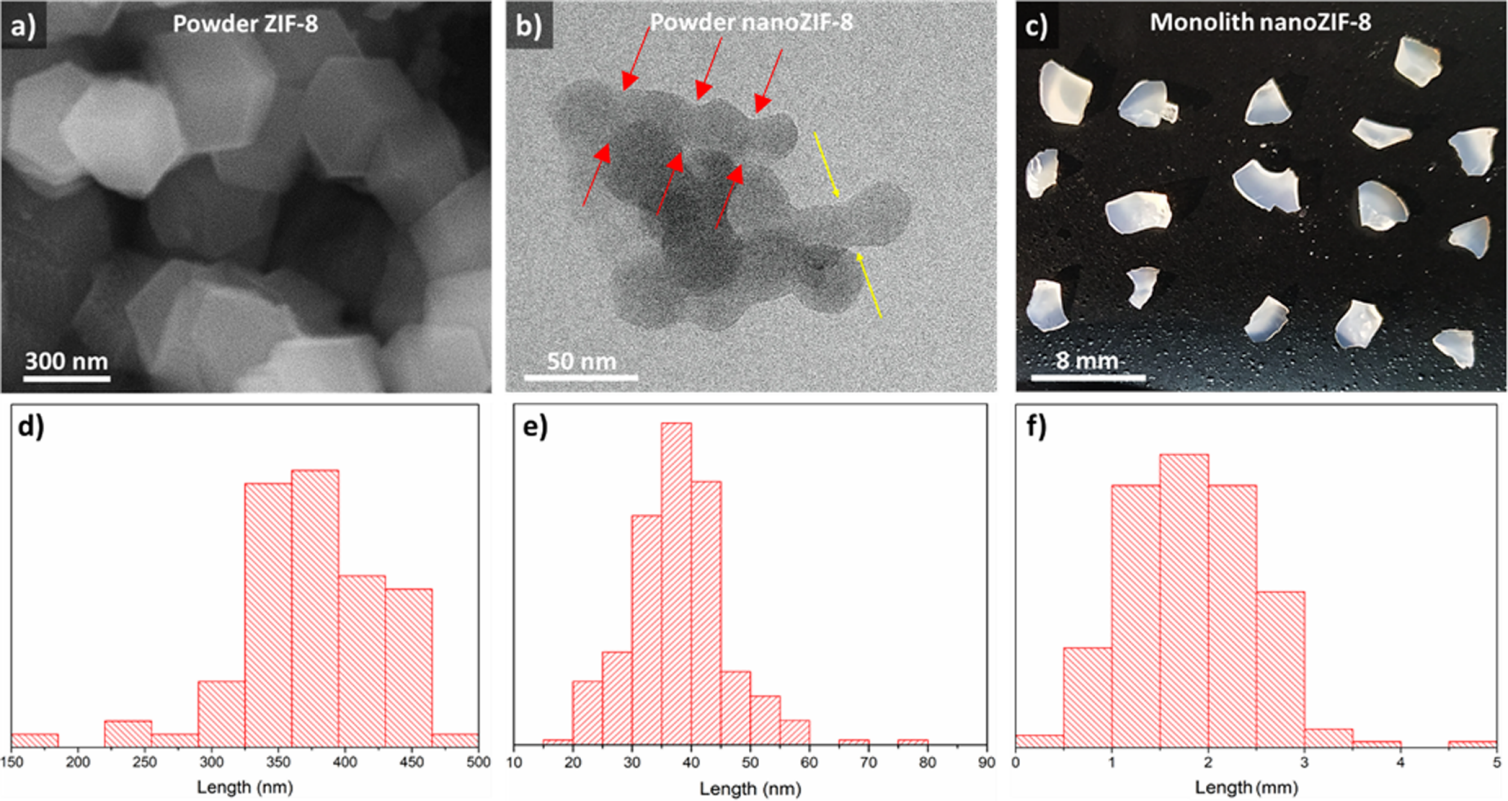
Representative images for different ZIF-8 samples: (a,d) SEM image and crystal size distribution for _powder_ZIF-8. (b,e) TEM image and crystal size distribution for _powder_nano_ZIF-8; here, the yellow arrows indicate GBs between crystals. The red arrows indicate GBs among a set of crystallites forming a powder_nano grain; this case shows that for powder_nano sample, crystallites are highly constrained from the formation of tight GBs. This is probably due to self-assembly of smaller crystallites preferentially exposing (110) surfaces, as discussed in ref (62). (c,f) Optical image and monolith size distribution for _mono_nano_ZIF-8.

Figure 1 shows the X-ray diffraction (XRD) for _powder_ZIF-8, _powder_nano_ZIF-8, _mono_nano_ZIF-8, and the simulated pattern of SOD ZIF-8 (Zn_6_(2-methylimidazole)_12_.^75,76^_mono_nano_ZIF-8 has broader peaks in the XRD pattern due to the smaller crystal size of the primary particles of the sol–gel process, as reported previously, on the order of *ca.* 30–40 nm.^46^ While having the same structure, these three materials are considerably different in the macroscopic morphology (Figure 2a–c). _powder_ZIF-8 and _powder_nano_ZIF-8 have a representative grain size of 300–500 and 15–60 nm, respectively (Figures 2d,e and S1). The “Necks” between crystallites in Figure 2b (highlighted by yellow arrows) are GBs, showing that the _powder_nano_ZIF-8 is made of aggregates of tightly bound nanocrystallites. Such GBs for ZIF-8 were recently demonstrated by high-resolution TEM.^62^_mono_nano_ZIF-8 is represented by 1–3 mm dense and transparent pieces (Figure 2c,f). The transparency of _mono_nano_ZIF-8 suggests the absence of macroscopic pores and small primary particles.^46^ As this has been broadly described in the literature in other sol–gel systems, the fact that the ZIF-8 monolith is transparent and the light is not scattered is, indeed, a consequence of the non-existence of electron-density interfaces inside the body that is the material is a continuous phase without any macro- or mesoporosity. Moreover, both the N_2_ isotherms and the mercury porosimetry show limited values for macroporosity (*ca.* 0.01 cm^3^/g) compared to microporosity (*ca.* 0.53 cm^3^/g). As we have shown for purely microporous MOFs such as ZIF-8^46^ and HKUST-1,^77^ including HRTEM and HAADF, the monolithic phase is that of a polycrystalline and continuous material. Moreover, N_2_ adsorption characterization (Figures S2–S6) reveals improved volumetric characteristics of _mono_nano_ZIF-8, that is, higher surface (*S*_BET_) and cavity volume per unit volume (*V*_Tot_^b^) of the porous sample (Table 1), which is due to its higher density. Its high density, the crystallite size, and the transparency of the sample suggest that the monolith consists of a tight aggregate of very small crystallites, probably forming among them tight GBs of the kind observed in the _powder_nano_ZIF-8 sample (Figure 2b).

**Table 1:** N_2_ Adsorption and Hg Porosimetry Characterization of _powder_ZIF-8, _powder_nano_ZIF-8, and _mono_nano_ZIF-8 Samples

material	*S*_BET_ (m^2^/g)	*V*_Tot_^b^ (cm^3^/g)	ρ_b_ (g/cm^3^)	*S*_BET_ (m^2^/cm^3^)	*V*_Tot_^b^ (cm^3^/cm^3^)
_mono_nano_ZIF-8	1452	0.55	1.05	1525	0.57
_powder_nano_ZIF-8	1591	0.65	0.65	1034	0.42
_powder_ZIF-8	1616	0.69	0.35	566	0.24

It is essential to distinguish between the grain size measured by TEM and a coherent domain size (crystallite size) measured by Debye–Scherrer broadening of Bragg peaks. Based on the XRD refinement, the coherent domain size for the _powder_nano_ZIF-8 sample is about 30 nm (which is in acceptable agreement with TEM results of about 40 nm). The coherent domain size for the _mono_nano_ZIF-8 sample (note, we do not call it monocrystalline) is about 12 nm. The grain sizes measured by the TEM do not have to match domain sizes from the XRD. In this context, the nano-size grain is a single domain of about 30 nm. The monolith block consists of multiple packed domains of about 12 nm.

At the moment, we can only hypothesize on why the packing of nano-size crystallites decreases the size of the domain. The physical reason for this effect could be associated with the formation of domain boundaries as was observed recently for ZIF-8.^62^ Answering this question is outside of the scope of this work.

### 3.2 Intrusion–Extrusion Study

**Figure 3:**
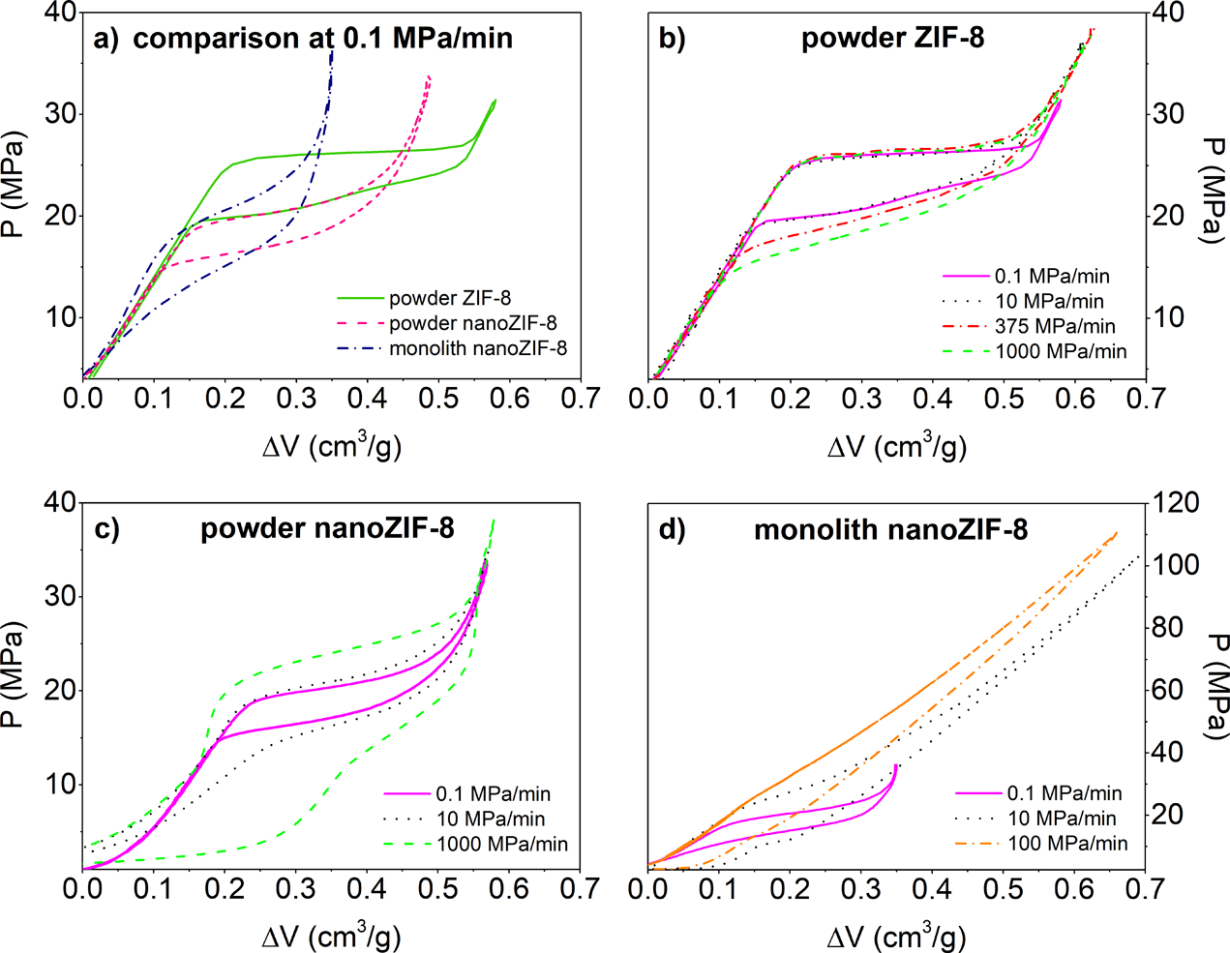
Room temperature *PV*-isotherms for the ZIF-8 + water system: (a) comparison between _powder_ZIF-8, _powder_nano_ZIF-8, and _mono_nano_ZIF-8 at 0.1 MPa/min compression rate; (b) _powder_ZIF-8, (c) _powder_nano_ZIF-8, and (d) _mono_nano_ZIF-8 at different compression rates.

Figure 3 shows the water intrusion–extrusion—*PV*-isotherm for ZIF-8 samples measured at different rates, from 0.1 to 1000 MPa/min. For _powder_ZIF-8, the shape of the hysteresis loop is of an intermediate type between the one for a molecular spring and a shock-absorber (Figure 3a). In other words, the hysteresis is not large enough to make the _powder_ZIF-8 + water system an efficient dissipator, but too large making it a poorly performing molecular spring. Moreover, while for some flexible materials, the hysteresis loop strongly depends on the compression–decompression (intrusion–extrusion) rate,^16^ this is not the case for _powder_ZIF-8, for which the *PV*-isotherm remains practically the same in a 5 order of magnitude rate span, even when extreme velocities of 1000 MPa/min are applied (Figure 3b). These kinetics of water intrusion–extrusion in seemingly small pores of ZIF-8 (*ca.* 10.8 and 3.4 Å flexible windows) is fascinating. While high-frequency operation has been demonstrated previously for mesoporous grafted silica + water systems,^8,13,14^ a similar feature is surprising for microporous ZIF-8, which under ambient conditions has a pore opening of only 3.4 Å and, perhaps, even more importantly may undergo reversible structural transition upon water intrusion–extrusion, known as a gate-opening effect.^78^ Results presented in Figure 3b suggest both a rapid intrusion–extrusion of water molecules into-from ZIF-8 as well as its fast framework response to an external stimulus, such as pressure. To gain a microscopic confirmation of the observed performance, *in operando* high-pressure neutron diffraction experiments were conducted on the _powder_ZIF-8 + water system.

### 3.3 *In Operando* Neutron Scattering Study

**Figure 4:**
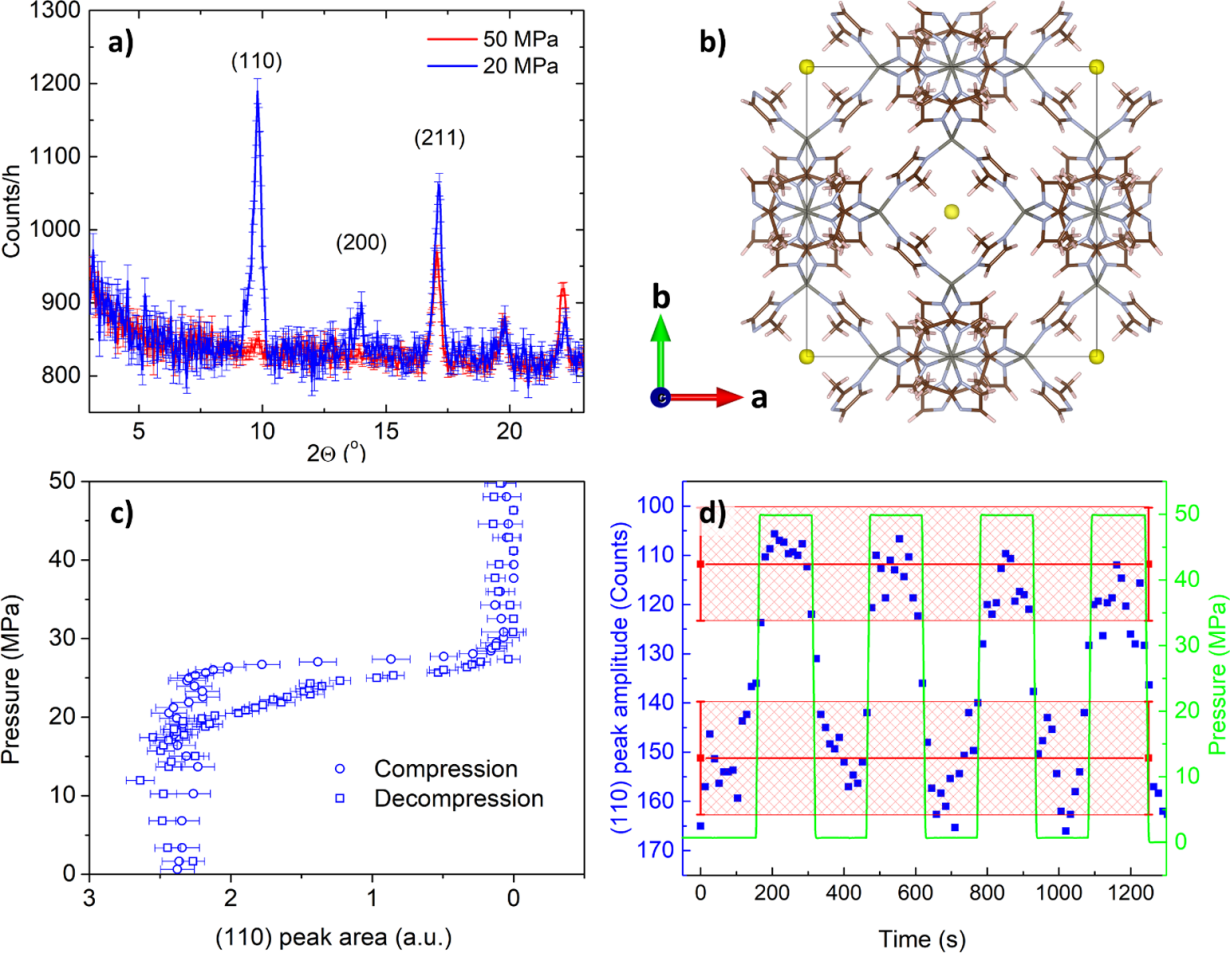
Neutron diffraction experiments for the _powder_ZIF-8 + water system: (a) diffraction pattern at 20 and 50 MPa (above and below intrusion, respectively); (b) Fourier map, showing the yellow isosphere to reflect the occupational difference between 20 and 50 MPa states; (c) pressure dependence of the area of the (110) reflection (note, the similarity with Figure 3a); and (d) intensity of the (110) reflection recorded upon dynamic compression–decompression cycling. Error bars indicate 1σ. Red shaded area indicates uncertainty.

Figure 4a shows the neutron powder diffraction patterns collected at the BT-1 diffractometer for the _powder_ZIF-8 + water system at 20 and 50 MPa. The pattern at 20 MPa corresponds to the empty framework of ZIF-8 below intrusion pressure, while 50 MPa corresponds to water-filled ZIF-8 as this pressure is well-above the intrusion pressure (Figure 3a). The obvious difference in two patterns is evident for the (110) Bragg peak at 9.85° (Figure 4a). The variation in this peak’s intensity is due to the contribution of the intruded water to the scattering factor, for which periodicity is induced by the crystal lattice. The contribution of water can be quickly extracted from the difference Fourier mapping applied to these patterns at 20 and 50 MPa, which reveals that the main difference is indeed in the occupancy of the pore of ZIF-8 (see yellow iso-surface in Figure 4b). Besides, Figure 4c shows the dependence of the (110) peak area on pressure, which perfectly mimics the *PV*-isotherm of the _powder_ZIF-8 + water system (Figure 3a). This relationship suggests indeed that the (110) peak can be used for *in operando* tracking of water molecules intrusion–extrusion into-from ZIF-8.

Figure 4d demonstrates the dependence of the (110) peak intensity on pressure upon compression–decompression cycles performed with 50 MPa/min ramp. One can clearly see that the variation of intensity closely follows the pressure ramp, confirming the good stability of the *PV*-isotherm of the _powder_ZIF-8 + water system at different compression–decompression ramps (Figure 3a) is indeed due to the fast kinetics of water molecules entering-leaving the cages of _powder_ZIF-8. Another important conclusion from this observation is that the well-known gate-opening effect of ZIF-8,^78^ related to the rotation of imidazolate linkers to ease intrusion of guest molecules in the ZIF-8 cavities, does not significantly hinder the intrusion–extrusion process within the timescale of the experiment. While this kinetics is impressive, the practical attractiveness of the _powder_ZIF-8 + water system remains questionable due to the intermediate hysteresis loop (neither molecular spring nor shock-absorber—Scheme 1).

### 3.4 Effect of Monolith Configuration and Crystal Size

To begin with, we focus on the intrusion/extrusion cycle at quasi-static conditions, that is, at low pressure scanning rates, 0.1 MPa/min. One notices two main differences between the intrusion/extrusion cycle in the powder_nano and monolith with respect to the powder one: (i) lower intrusion pressure and (ii) lower intrusion volume (Figure 3a). The former is explained by the already observed relationship between crystallite size, which is smaller in both _powder_nano_ZIF-8 and _mono_nano_ZIF-8, and intrusion pressure.^31,79^ The latter, we attribute to the tighter packing of the crystallite in both _powder_nano_ZIF-8 and _mono_nano_ZIF-8 samples, which prevents the sizable expansion of ZIF-8 accompanying liquid intrusion, as recently reported.^80^ This effect is discussed in detail in the theoretical Section 3.5. Additionally, the tight packing, especially in the monolith sample, might slow down or prevent percolation of water in the core of the sample, thus limiting the amount of ZIF-8 which is actually intruded during a cycle. The atomistic origin of this phenomenon is also discussed in the theoretical section. Finally, one cannot exclude a possible amorphization of the surface layer of crystals, which will result in intrusion volume reduction for _powder_nano_ZIF-8 and _mono_nano_ZIF-8 compared to _powder_ZIF-8. In Figure S7, *PV*-isotherms for these samples are compared by normalizing the volume change with the initial crystallographic volume. One can appreciate that compensation for the unit cell size does not influence the differences in the intrusion volume described above.

Another remarkable difference between the three samples is the slope of the intrusion branch of the *PV*-cycle. One notices that the branch of the *PV*-cycle corresponding to intrusion is (almost) flat in the case of _powder_ZIF-8, suggesting that intrusion takes place at a well-defined pressure, while it acquires a slope for the _powder_nano_ZIF-8 sample, which further increases in the case of _mono_nano_ZIF-8. The dependence of the slope of the intrusion branch on the arrangement of the ZIF-8 sample is reported here for the first time and suggests that in _powder_nano_ZIF-8 and _mono_nano_ZIF-8, intrusion takes place over a range of pressures. Atomistic simulations help us to understand and explain the origin of this behavior, as discussed in Section 3.5.

**Figure 5:**
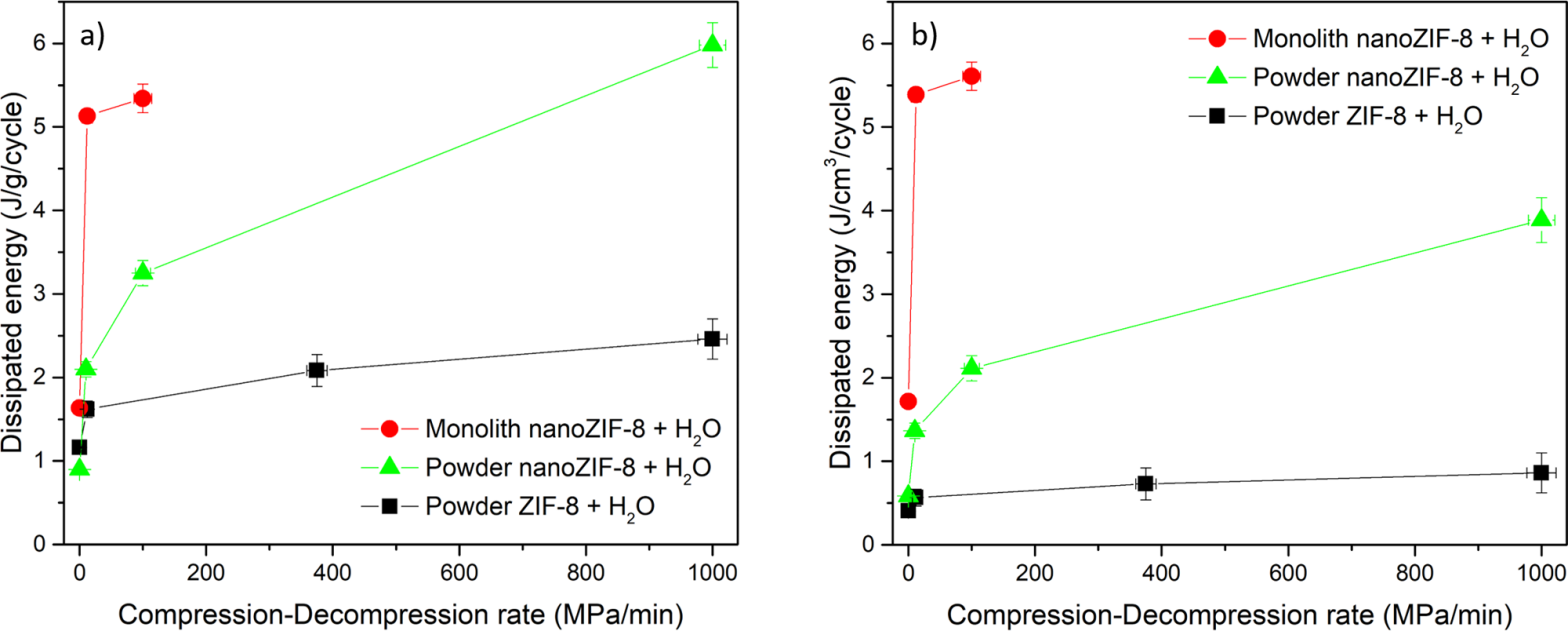
Amount of dissipated mechanical energy per intrusion–extrusion cycle for _powder_ZIF-8, _powder_nano_ZIF-8, and _mono_nano_ZIF-8 depending on the compression–decompression rate: (a) per unit of mass and (b) per unit of volume. Note that bulk density of the powder is used to calculate the volumetric energy density for the cases of _powder_ZIF-8 and _powder_nano_ZIF-8 cases.

What is very interesting and technologically appealing is the dependence of the dynamic response of the “ZIF-8 + water” system on the compression–decompression rate on the crystal size (powder vs powder_nano—Figures 3b vs 3c) and compactness of the crystallite aggregate (nano-powder vs nano-monolith configuration—Figure 3c vs 3d). The effect of crystal size of ZIF-8 on the intrusion–extrusion behavior is somehow expected from previous studies,^31,79^ while the effect of crystallite aggregate compactness—monolith configuration—is unexpected and, to the best of our knowledge, has never been presented previously. More specifically, in the case of monolith configuration, increasing the speed of cycling effectively transforms a poorly performing molecular spring into an effective shock-absorber (Figure 3d). The technological potential of the monolith configuration versus powder cases is illustrated in Figure 5, showing the amount of dissipated mechanical energy per intrusion–extrusion cycle (i.e., the area of the hysteresis loop of Figure 3). Here, one notices a drastic difference in the dependence of dissipated energy on the compression–decompression rate for the three samples. For _mono_nano_ZIF-8, increasing the ramp from 0.1 to 10 MPa/min results in a more than threefold increase in dissipated energy, which, despite the reduction of the intruded volume, is more than three times higher compared to _powder_ZIF-8 under similar conditions (Figure 5a). Moreover, considering the higher density of _mono_nano_ZIF-8, the volumetric dissipated energy density improved more than 1 order of magnitude compared to _powder_ZIF-8 (Figure 5b). It also interesting to note that at velocities of around 10 MPa/min _mono_nano_ZIF-8 demonstrated two-step extrusion, which was noted earlier for _powder_ZIF-8 depending on the compression rate, temperature^81^ and perhaps due to the interplay between the extrusion process and opening-the-gate effect or the extrusion of water from the GB. Such a two-step extrusion is also evident for _powder_nano_ZIF-8 at high compression–decompression rates (Figure 3b). The mechanism behind this behavior is outside of the scope of this paper and will be explored in the future.

We note, that in this work, we intentionally avoid relating the compression–decompression rates to operational frequencies because there is a key difference between a high-frequency experiment versus experiment with a high compression–decompression rate, which is the dwell time after intrusion/extrusion. This pause may play a key role, providing time for system relaxation. Therefore, linking compression–decompression rate with frequency may be misleading.

The reported results suggest that both crystal size and monolith configuration have a drastic effect on the dynamic intrusion–extrusion performance of water into-from ZIF-8: both smaller crystallites and the monolith configuration help increasing hysteresis with the pressure scanning rate. The effect of the monolithic configuration, however, is more pronounced from the energetic point of view. Indeed, it can be seen that with the monolith configuration, dissipated energy of 5.3 J g^–1^ cycle^–1^ can be reached at compression–decompression rates of 100 MPa/min (Figure 5a). _powder_nano_ZIF-8 requires more than 8 times higher compression–decompression rate to reach similar dissipated energy (Figure 5a). Moreover, volumetric energy density of the monolith configuration is more than 5 times higher compared to _powder_nano_ZIF-8 and _powder_ZIF-8 at 100 MPa/min compression–decompression rate. Remarkably, powder samples cannot match the high volumetric energy density of the monolith configuration even at the very high pressure scanning rates. Therefore, while crystal size is indeed important, the monolith configuration clearly introduces additional benefits to the dissipation capabilities of ZIF-8. In other words, present findings suggest a novel tuning strategy that can be exploited to enhance the energy dissipation of ZIF-8 samples, which might open novel technological applications of this material and/or other MOFs. To conclude this paragraph, it is worth remarking that even though the enhancement of dissipated energy due to crystal size is less pronounced as compared to monolith configuration, the benefit of the crystal size-approach in lower intrusion–extrusion pressures (Figure 3c), which may be beneficial for certain applications, such as shock-absorbers.

In the rest of this section, we propose possible mechanisms underlying the effect of monolith configuration on the intrusion–extrusion dynamics, which are then expounded using atomistic simulations in the next section. In order to exclude the possible differences in hydrophilicity of the samples, thermogravimetric experiments were conducted for all three samples after maintaining them under control humidity of 90% for 24 h—Figure S8. There is no noticeable difference between _powder_ZIF-8 and _powder_nano_ZIF-8, which both decompose at temperatures above 600 °C. However, it can be seen that _mono_nano_ZIF-8 exhibits some mass loss at around 250 °C. This is related, as suggested previously, to the release of unreacted imidazolates.^82^ The fact that this mass loss is not related to the adsorbed humidity can be clearly seen when comparing thermograms for the sample subjected to 90% and the sample which was activated under 100 °C and vacuum immediately prior to the thermogravimetric measurement—Figure S9. One can see from this figure that this sharp step is present for both samples. This observation suggests that there indeed could be some contribution from the hydrophilic defects; however, it is unlikely to be the main factor explaining the difference in the intrusion–extrusion volume between the three samples of ZIF-8. First of all, a 14% decrease of the intrusion–extrusion volume is observed for _powder_nano_ZIF-8 compared to _powder_ZIF-8, while the thermogravimetric experiment is similar for these two samples, within less than 1% tolerance (Figures S8 and S10). On the other hand, N_2_ adsorption experiments seem to correlate with the intrusion–extrusion experiments more clearly—Figures S10 and S11. TG results suggest that the contribution from the hydrophilic defects is unlikely to be the main factor explaining the difference in the intrusion–extrusion volume between the three samples of ZIF-8.

Considering that the intrusion–extrusion process in itself is rapid (Figures 3b and 4d), it is reasonable to assume that the observed differences in the dynamic hysteresis for monolithic and powder ZIF-8 are due to the arrangement of their grains (densely packed monolith vs fine powder). As we mentioned in Section 3.1, the high density, the crystallite size, and the transparency of the monolith suggest that it is composed of tightly packed crystallites. In this case, it is expected that a longer time is required for water to percolate through, in particular to reach crystallites at the core of the monolith, which causes higher intrusion pressure upon forced compression, as well as lower pressure of spontaneous extrusion upon rapid decompression (Figure 3c). This contrasts with _powder_ZIF-8, with a much higher external surface directly in contact with the bulk liquid, which speeds up the process. Combining this rather straightforward phenomenon with a highly responsive intrusion–extrusion process (Figures 3b and 4d) allows for the tunability of dynamic hysteresis of heterogeneous lyophobic systems depending on the density of the grains of the porous material (Figures 3d and 5). This provides an opportunity for the development of energy dissipators with frequency-dependent performance. Moreover, it is useful for the triboelectrification phenomenon, which was previously demonstrated to be linked with mechanical and thermal energy hysteresis in the intrusion–extrusion cycle^15,16^ and will be explored in more detail in upcoming works.

A final remark on the stability of the sample upon repeated liquid intrusion/extrusion is in order as it concerns the potential technological relevance of the monolith or, in general, crystallite packing as a strategy to enhance energy dissipation. Stability of porous materials upon intrusion–extrusion cycling is a challenge, and monolith stability is no exception. Nevertheless, after intrusion–extrusion cycling tests (overall 36 cycles), a good repeatability of the results was evident. The samples maintained their size (Figures S12), even though, some cracks were observed—Figure S13. Additionally, for the stability verification, we compared the intrusion–extrusion cycles at 0.1 MPa/min recorded before and after dynamic cycling and a good reproducibility was evident—Figure S14.

We expect that the obtained effect of monolith configuration will not be specific to ZIF-8, and other MOFs such as ZIF-67,^81^ MAF-7,^83^ ZIF-71, and MAF-6^84^ can be considered to enhance their energy dissipation capabilities. This will be explored in upcoming works.

### 3.5 MD Simulation Study

Simulations were performed to validate the hypotheses proposed to explain the differences in the intrusion–extrusion characteristics of powder, powder_nano and monolith samples, that is, to assess how the structural effects of tight packing of ZIF-8 might alter the intrusion/extrusion dynamics. We focused on GBs: indeed, as mentioned above, _powder_nano_ZIF-8 and, in particular, _mono_nano_ZIF-8 are characterized by a high density of GBs, more GBs per crystallite than the powder sample. Moreover, smaller crystallites show a rhombic dodecahedral shape (Figure 2b) versus the cubic shape typical of larger crystallites (Figure 2a). Previous work has shown that the (110) surface exposed by the former allows the formation of tightly bound, “locked”, GBs, which are not observed for the (100) surface, exposed by the latter.^62^ In the following, we show that tight GBs, solely formed for samples containing smaller crystallites—_powder_nano_ZIF-8 and _mono_nano_ZIF-8—may slow down the percolation of water in the interior of the monolith and/or prevent liquid intrusion into ZIF-8.

GBs are complex structures and some *a priori* information or assumption is needed for their modeling. In the case of ZIF-8, a previous study^62^ has revealed that ZIF-8 nanocrystals of ∼85 nm, a size comparable with those measured in powder_nano and mono_nano samples, have a rhombic dodecahedral shape consistent with images reported in Figure 2. These crystals expose (110) surfaces, which can be either “zigzag” or “armchair” terminated, the latter being the one experimentally observed. Quoting ref (62) “ZIF-8 crystals were “locked” at (110) interfaces after attachment, [...], indicating that (110) surfaces are particularly important for self-assembly”. Given this solid experimental evidence, here, we focused on an armchair-terminated (110) GB. Given the complexity of the system, we decided to use an *ab initio* approach, performing DFT calculations as described in detail in Section 2.2.6.

**Figure 6:**
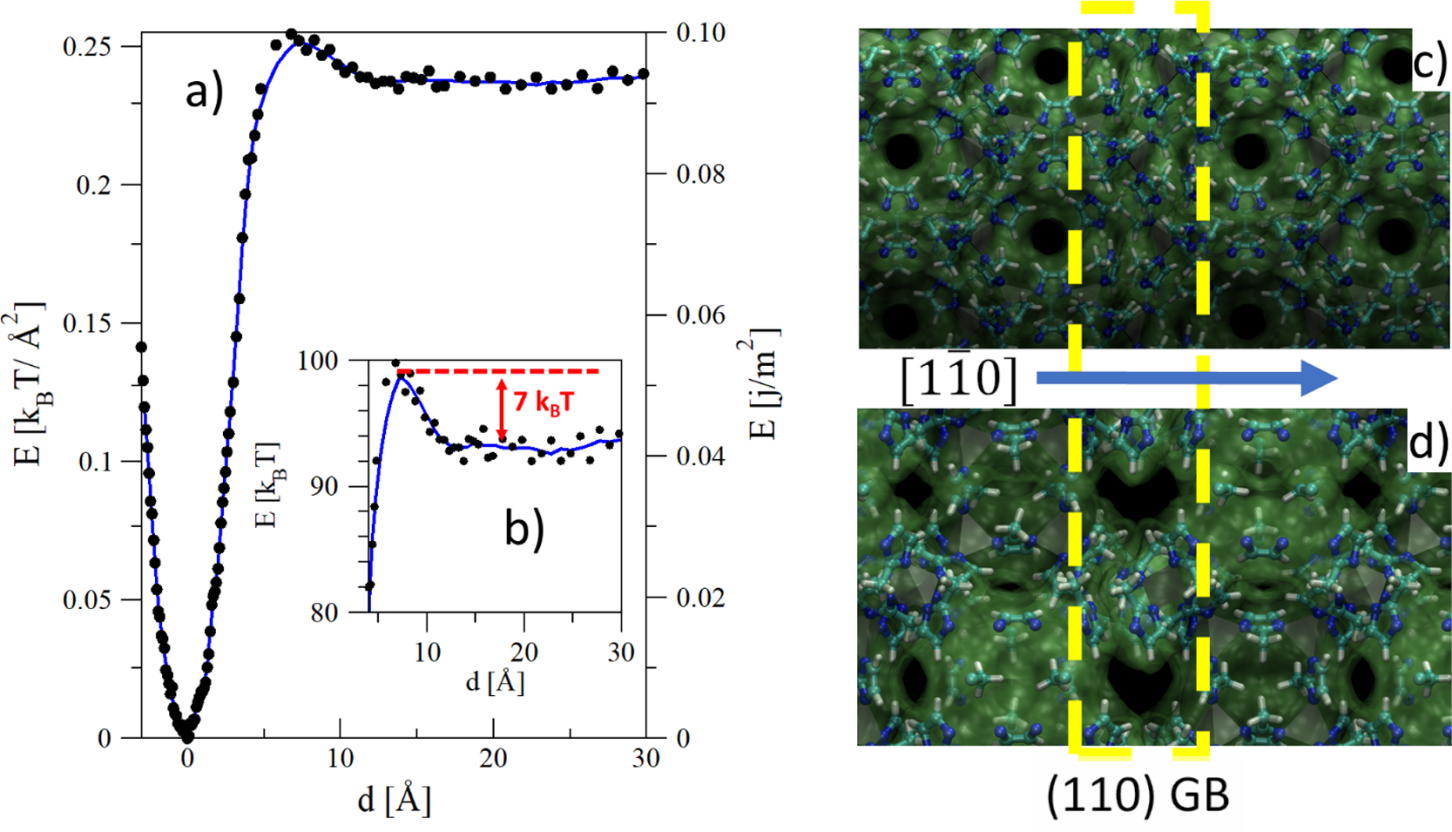
(a) Energy profile of the ZIF-8 (110) GB as a function of the distance between the two crystallites. The profile is characterized by a deep minimum and a barrier at ∼5 Å. (b) Zoom in on the barrier region. In panel (b), the energy profile is reported in *k*_B_*T* for a GB of ∼392 Å^2^ of contact area, corresponding to the smallest possible ZIF-8 (110) GB. The height of the barrier with respect to the energy plateau at larger distances 7 *k*_B_*T*, that is, 7 times the thermal energy available at room conditions, suggesting, according to the Arrhenius law, that a close approach between two crystallites is energetically non-trivial, especially for large crystallites. (c,d) Images illustrating the GB from two different points of view. The crystallites and the GB are shown by both a stick-and-ball representation of the atoms and the surface enveloping the atoms obtained by a probe particle^85^ of 3.0 Å of diameter, approximately corresponding to the characteristic size of water in several classical force fields.^74^

To start with, we accurately studied the structure of the GB. Here, we refrained from performing a simple structural optimization of the GB as the potential of such a complex system might present roughness, which could trap the structure in local minima, at a distance between the two crystallites different from the equilibrium one. Rather, we searched for the lowest energy structure as a function of the distance *d* between the two ZIF-8 crystallites forming the GB, exploring a broad ∼35 Å distance range. The profile of the energy of the GB versus the distance is reported in Figure 6a, where distance *d* = 0 Å has been arbitrarily fixed in correspondence of the minimum of the GB energy. Panels d and e of the same figure show two views of the GB, highlighting how tight the two crystallites are in the stable configuration. Concerning the energy profile, one interesting feature is the energy maximum at ∼5 Å, amounting to a ∼7 *k*_B_*T* barrier to allow two facing ZIF-8 crystallites to grow along the [110] direction to reach the most stable configuration (Figure 6b). Of course, such a barrier increases with the area of the facing crystallites, quickly exceeding the thermal energy or other forces that may push the system beyond the barrier, for example, the reduction of free energy along the growth of crystallites, which can help to tightly bind crystallites during the self-assembly process. This observation might explain why this tight binding is observed only for the powder_nano (Figures 2b and S1) and, possibly, the monolith samples and not for the regular powder with larger crystallites.

**Figure 7:**
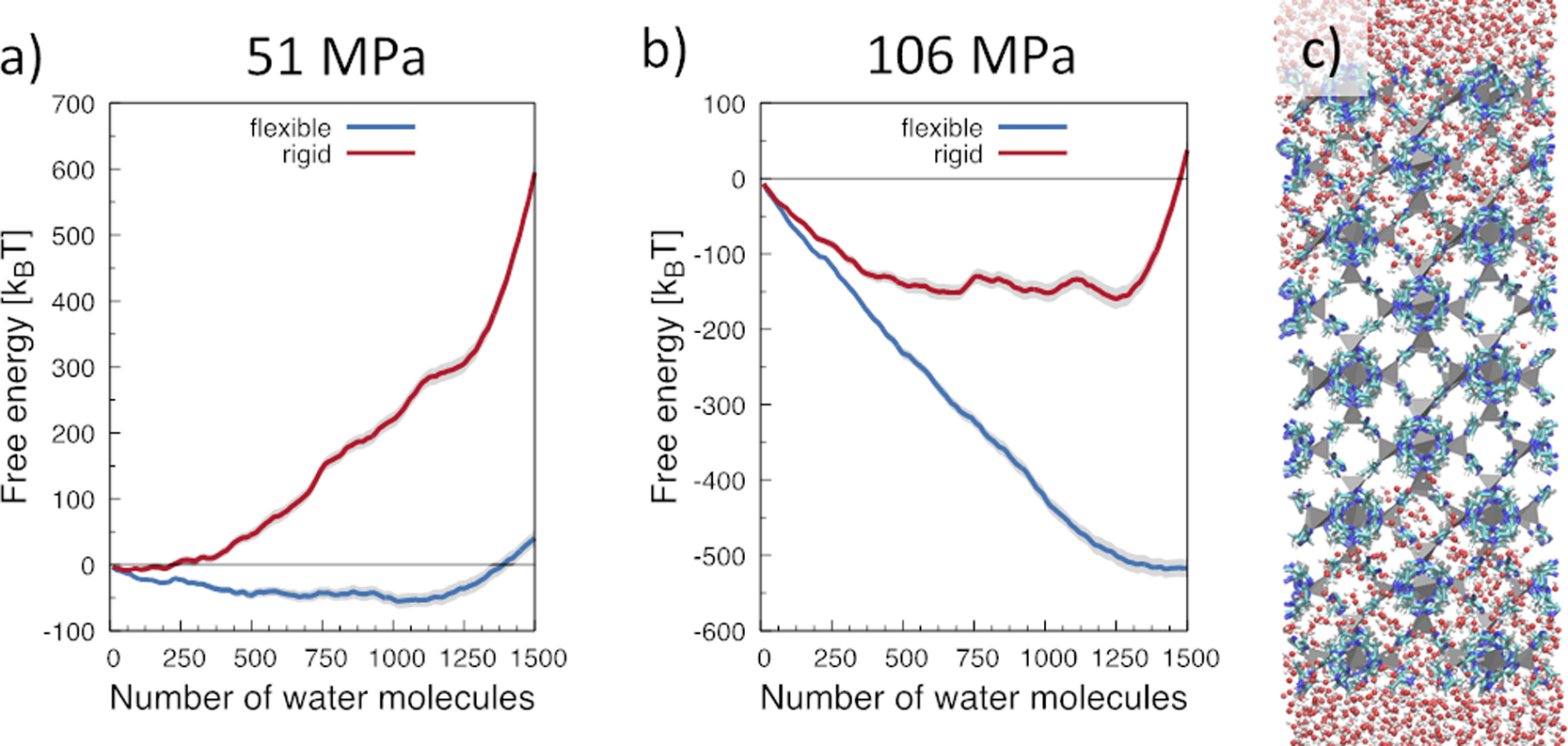
(a,b) Free energy profile of the ZIF-8 + water sample as a function of the number of water molecules in the MOF slab, shown in panel c in a partially filled configuration. The gray shadow beneath the solid lines represents the error on the estimation of the free energy (see the Supporting Information). In panel (a), we report the free energy profiles at the computational intrusion pressure of the flexible framework, 51 MPa. Indeed, one can notice that in these conditions the free energy of the filled state (1050 water molecules in the framework) is lower than the empty one. At this pressure, on the contrary, for the rigid framework the lowest free energy state corresponds to the empty ZIF-8 framework. At 106 MPa, the lowest free energy state is the filled one also for the rigid framework case, indicating that at this pressure intrusion also takes place in this sample. Due to liquid and lattice compressibility, at 106 MPa, the water molecules in the filled ZIF-8 slab are more than at 51 MPa.

As mentioned above, the tight binding of grains might have two consequences: (i) it may prevent the expansion of grains during intrusion, the latter being a phenomenon recently reported by some of the authors of the present work,^80^ and (ii) slow down percolation of water, preventing, in practice, the liquid to reach ZIF-8 crystallites in the core of the monolith. Let us analyze in detail how these two mechanisms may affect intrusion, starting with the tight binding among crystallites preventing or limiting their expansion and the consequences on the wetting of the ZIF-8 cavities. Figure 7a,b compares the free energy profiles of liquid intrusion in a flexible and rigid ZIF-8 framework at 51 and 106 MPa and the computational intrusion pressures of the flexible and rigid frameworks (Figure 7c), respectively. We remark that intrusion free energy profiles have been determined using the RMDs approach, with ZIF-8, water and their interaction modeled by a classical force field, an approach that has been successfully employed in previous works^80,86^ (see Section 2.2.6 and the Supporting Information for further details). In the rigid framework, atoms are still allowed to move. In particular, the imidazolate is allowed to rotate to ease liquid intrusion through the six-member ring windows (see Figure 6d), but the crystallite is globally prevented to expand/compress (see Section 2.2.6). The intrusion pressure can be determined by identifying the value at which the free energy corresponding to full wetting of the ZIF-8 slab, that is, when all cages of the computational sample (Figure 7b) are filled, is lower than that of the empty slab. Due to the liquid compressibility, the actual number of water molecules in the filled slab changes with pressure. It is seen that despite the expansion upon complete intrusion being apparently small, ∼0.06 Å per unit cell,^80^ rigidity increases the intrusion pressure by , bringing it from 51 mPa, as predicted for the flexible ZIF-8, to 106 mPa, as determined for the rigid one. Indeed, this effect of flexibility on the intrusion pressure may explain the surprisingly low value of  of ZIF-8  versus more rigid porous materials of comparable porous size and hydrophobicity, such as MFI ()^87^ and TON (),^88^ and with respect predictions of the Young–Laplace equation for ZIF-8:  ( = 72.8 mN/m is the water surface tension,  the Young contact angle, here set to the apparent experimental value of 130°, and *r* = 1.7 Å the radius of the six-member ring apertures allowing intrusion). Our simulations suggests that the small ∼0.06 Å expansion of the lattice parameter crucially reduces the intrusion pressure of ZIF-8 and that, on the contrary, hindrance of lattice expansion, as the one imposed by tight GBs, may severely limit the number of crystallites that can be intruded in the nanopowder and, especially, in the monolith. Of course, we neither expect that tight GBs do completely prevent crystallites expansion, nor that in an experimental sample all crystallites are compressed to a level of tightness corresponding to the GB equilibrium distance. GB equilibrium distance is the energetically favored configuration of a GB but it is well known that crystallization is controlled by a subtle balance of thermodynamics and kinetics factors.^a^ We expect that GBs impose a partial limitation and/or a hindrance on expansion and that these are more severe in the densely packed monolith than in the powder_nano sample and absent in the standard powder, made by cubic crystallites lacking extended (110) surface allowing the formation of tight GBs. This, possibly, results in intrusion taking place in a pressure range rather than at a well-defined value, depending on the presence of crystallites at various degrees of compression in the powder_nano and mono_nano samples. This will result in the slope of the intrusion branch of the *PV*-cycle of the powder_nano and mono_nano samples shown in Figure 3a. The slope of the latter is larger than that of the former, consistently with the higher density of tight GBs one expects in the case of the monolith. The most compressed grains, probably those laying in the core of the monolith or in more tightly bound crystallites of the _powder_nano_ZIF-8 sample (Figure 2b), might be completely prevented to be intruded in the relevant pressure range, which explains the sizable reduction of intruded volume of the ZIF-8 configuration.

**Figure 8:**
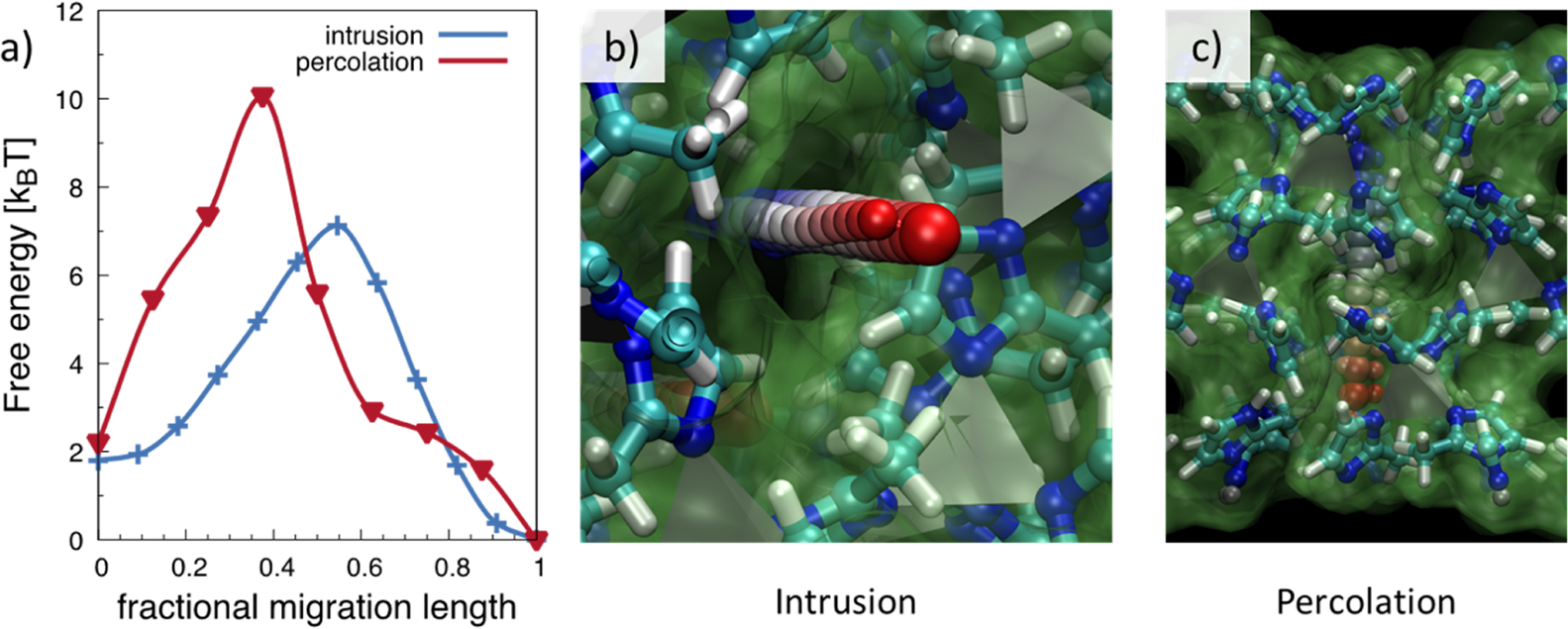
(a) Energy profile of intrusion (blue) and percolation (red) of a water molecule in ZIF-8 and along the (110) GB, respectively. In panel (b,c) are reported the corresponding paths. Panel (a) shows that, on the contrary of the trend with small non-polar molecules,^62^ water percolation along a tight GB requires to overcome a higher barrier with respect to intrusion.

Let us now focus on the effect of a tight GB on water percolation in the monolith sample. Of course, this effect is relevant if the characteristic times of water percolation, , and water intrusion, , are comparable. The characteristic time of diffusional processes is associated to the presence of energy barrier molecules must overcome along their path, the energy barrier associated to the crossing a six-member ring windows, , and the tangential diffusion along GBs, , in the case of intrusion and percolation, respectively. Percolation and intrusion times are associated to the corresponding barriers via an Arrhenius-like equation, , where the pre-exponential factor  is the intrinsic time it takes for the system to complete the process in absence of the barrier.^89^ Given the exponential dependence on the barrier,  mainly affects the intrusion or percolation times, and the one that depends the most on the confinement conditions. To evaluate the effect of tight GBs in limiting intrusion because of hindered of percolation, we computed the intrusion and percolation barriers of a single water molecule in the (*ab initio*) computational sample containing the (110) GB at the equilibrium distance. We recognize that this is a simplistic representation of water intrusion in ZIF-8 and percolation through the monolith, where, for example, there is more than one water molecule per ZIF-8 cavity or in the GB. However, the very high computational cost of the calculations necessary to compute intrusion and percolation barriers forced us to limit the complexity of the computational model. Nevertheless, we believe that these calculations reveal interesting phenomena that help interpreting the experimental results and, possibly, inspire further theoretical and experimental investigations. In Figure 8a, we report the energy profile of the intrusion and percolation energies, together with the corresponding transition paths (Figure 8b,c). Contrary to previous hypotheses, present results show that the percolation barrier of water in a (110) ZIF-8 GB at the equilibrium distance is slightly higher than the intrusion barrier. We believe that this is due to a combination of two factors: (i) at the equilibrium distance, the largest apertures along the GB are not sizably bigger than the six-member ring windows (see Figure 6c); moreover, (ii) the interactions between water and imidazolate molecules at GBs is stronger than in the ZIF-8 cavities, namely, water can form hydrogen bonds with nitrogen atoms because at the GB lone pairs of this chemical species are not involved in bonds with Zn.

The reduced percolation of water might completely prevent intrusion in the ZIF-8 crystallites laying at the core of the monolith sample. Though this effect is expected to be less problematic in nano_powder because ZIF-8 crystallites are in direct contact with bulk water, Figure 2b shows that a significant fraction of the surface of crystallites in this sample is engaged in the formation of tight GBs (see also Figure S1). Thus, also in this sample, the direct contact with bulk water is significantly reduced with respect to the standard powder sample.

Summarizing, atomistic simulations support the hypotheses proposed on the basis of experimental results to explain the dependence of intrusion/extrusion characteristics at quasi-static and high scanning rates on the type of aggregation of ZIF-8, _powder_ZIF-8, _powder_nano_ZIF-8, and _mono_nano_ZIF-8. Tight GBs present in the powder_nano and mono_nano can be seen as “extended” surface defects altering the *PV*-characteristics of these samples with respect to the standard powder one alluded in the literature.^79^ One cannot exclude that some of the observed differences between the studied ZIF-8 samples are also related to hydrophilic surface layer, effect of which is more predominant for samples with smaller crystal size.

## 4 Conclusions

Herein, we show that the dynamic hysteresis of a non-wetting liquid intrusion–extrusion process can be drastically affected by a macroscopic grain arrangement of a porous material. The concept is demonstrated by comparing the dynamic hysteresis of water intrusion–extrusion into-from a powder, hydrophobic ZIF-8 MOF, versus its monolithic highly dense analogue. We found that by changing the macroscopic morphology and arrangement of ZIF-8 from a fine powder to compact monolith, it is possible to change the intermediate intrusion–extrusion performance (nor molecular spring nor shock-absorber) into a desirable shock-absorber type with more than 1 order of magnitude enhancement of dissipated energy per cycle. The experimental results are supported by atomistic simulations and pave the way for a new strategy for tuning energy performance and applicability of molecular springs and nano-shock absorbers.

## Supporting Information Available

Characterization of _powder_nano_ZIF-8, gas adsorption characterization, BET area calculation using BETSI, intrusion–extrusion isotherms normalized per initial volume, experiments related to intrusion volume differences, stability of monolith samples, string method, and free energy calculations (PDF)

## Footnotes

a The thermodynamically stable outcome of a crystallization is a single crystal, which presents the lower surface/volume ratio, hence pays the lowest surface energy *penalty*. Producing a single crystal, however, is notoriously non-trivial, indicating that, in practice, crystallization is determined by both the thermodynamics and kinetics of the process.
