# Supplementary material for: Turning Molecular Springs into Nano-Shock Absorbers: The Effect of Macroscopic Morphology and Crystal Size on the Dynamic Hysteresis of Water Intrusion–Extrusion into-from Hydrophobic Nanopores
Source: ACS Appl Mater Interfaces. 2022 Jun 3;14(23):26699–713. doi: 10.1021/acsami.2c04314 (PMC9204699; doi:10.1021/acsami.2c04314)
Supplement: Supplementary file 1 — am2c04314_si_001.pdf [file am2c04314_si_001.pdf]

Supporting information

Turning molecular spring into nano-shock absorber: the effect of macroscopic morphology and crystal size on the dynamic hysteresis of water intrusion-extrusion into-from hydrophobic nanopores

Paweł Zajdel,[1] David Gerard Madden,[2] Robin Babu,[2] Marco Tortora,[3] Diego Mirani,[4] Nikolay Nikolaevich Tsyrin,[5] Luis Bartolomé,[6] Eder Amayuelas,[6] David Fairen-Jimenez,[2] Alexander Rowland Lowe,[7] Mirosław Chorążewski,[7] Juscelino B. Leao,[8] Craig M. Brown[8,9], Markus Bleuel,[8,10] Victor Stoudenets,[5] Carlo Massimo Casciola,[3] María Echeverría,[6] Francisco Bonilla,[6] Giulia Grancini,[4] Simone Meloni,[11] Yaroslav Grosu[6,7,*]

[1] Institute of Physics, University of Silesia in Katowice, 75 Pulku Piechoty 1, 41-500 Chorzow, Poland

[2] The Adsorption & Advanced Materials Laboratory (A²ML), Department of Chemical Engineering & Biotechnology, University of Cambridge, Philippa Fawcett Drive, Cambridge CB3 0AS, UK

[3] Dipartimento di Ingegneria Meccanica e Aerospaziale, Sapienza Università di Roma, via Eudossiana 18, 00184 Rome, Italy

[4] Department of Chemistry & INSTM University of Pavia, Via Taramelli 14, Pavia I-27100, Italy

[5] Laboratory of Thermomolecular Energetics, National Technical University of Ukraine "Igor Sikorsky Kyiv Polytechnic Institute", Pr. Peremogy 37, 03056 Kyiv, Ukraine

[6] Centre for Cooperative Research on Alternative Energies (CIC energiGUNE), Basque Research and Technology Alliance (BRTA), Albert Einstein 48, 01510 Vitoria-Gasteiz, Spain. ygrosu@cicenergigune.com

[7] Institute of Chemistry, University of Silesia in Katowice, Szkolna 9, 40-006 Katowice, Poland

[8] NIST Center for Neutron Research, National Institute of Standards and Technology, Gaithersburg, Maryland 20899, United States

[9] Chemical and Biochemical department, University of Delaware, Newark, Delaware 19716, USA

[10] Department of Materials Science and Engineering, University of Maryland, College Park, Maryland 20742-2115, United States

[11] Dipartimento di Scienze Chimiche e Farmaceutiche (DipSCF), Università degli Studi di Ferrara (Unife), Via Luigi Borsari 46, I-44121, Ferrara, Italy

1. **Characterization of $_{powder_nano}$ZIF-8** **2**
2. **Gas adsorption characterization** **3**
3. **BET area calculation using BETSI** **4**
4. **Intrusion-extrusion isotherms normalized per initial volume** **8**
5. **Experiments related to intrusion volume differences** **9**
6. **Stability of monolith samples** **11**
7. **String method** **12**
8. **Free energy calculations** **12**
9. **References** **15**

1. Characterization of $_{powder_nano}$ZIF-8

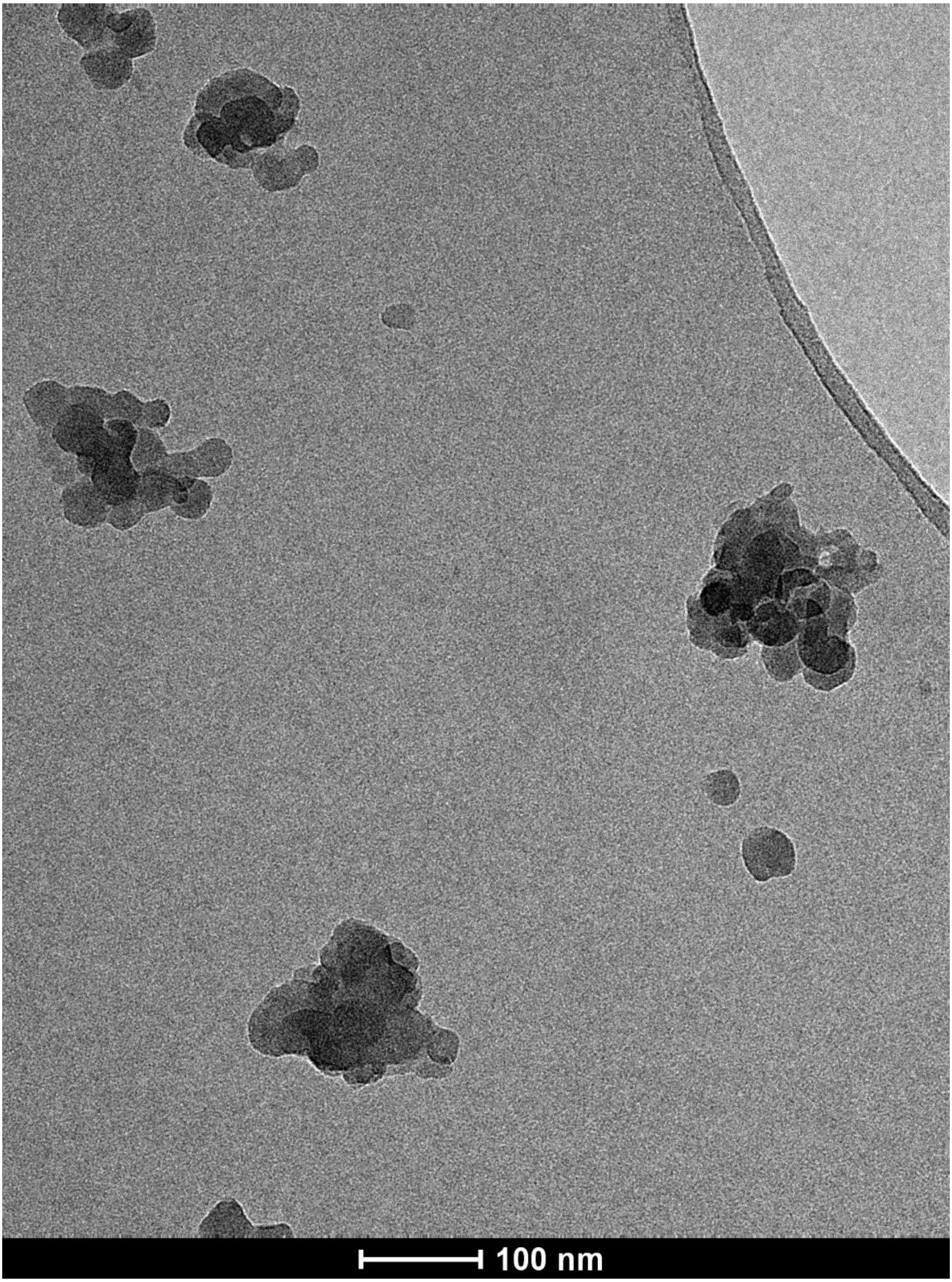

Figure S1. Representative TEM image of $_{powder_nano}$ZIF-8

2. Gas adsorption characterization

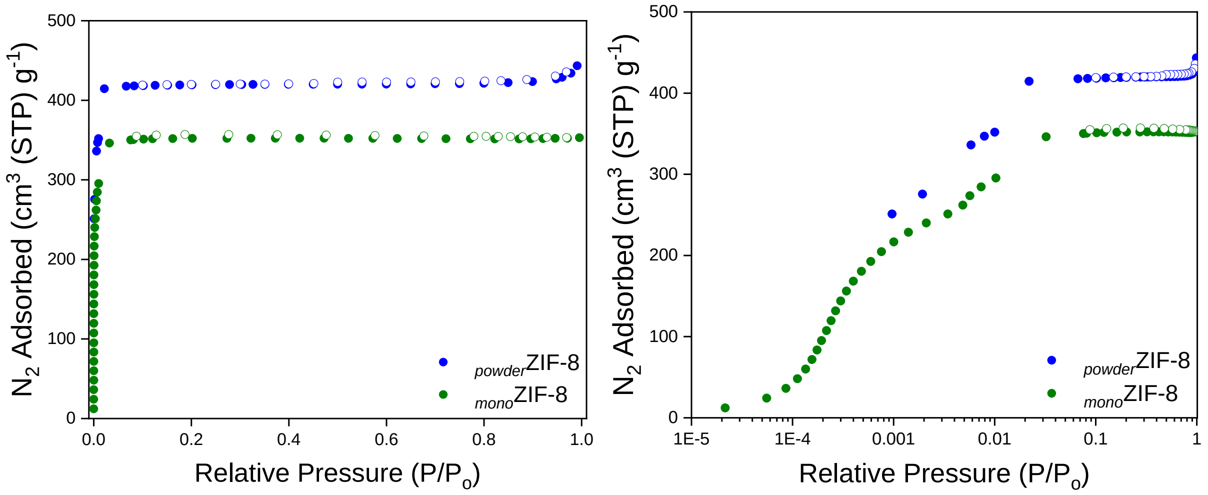

Figure S2. Gravimetric 77 K N_2 isotherms for $_{powder}$ZIF-8 and $_{mono}$ZIF-8 in (Left) linear and (Right) logarithmic scale. Closed symbols represent adsorption, open symbols represent desorption.

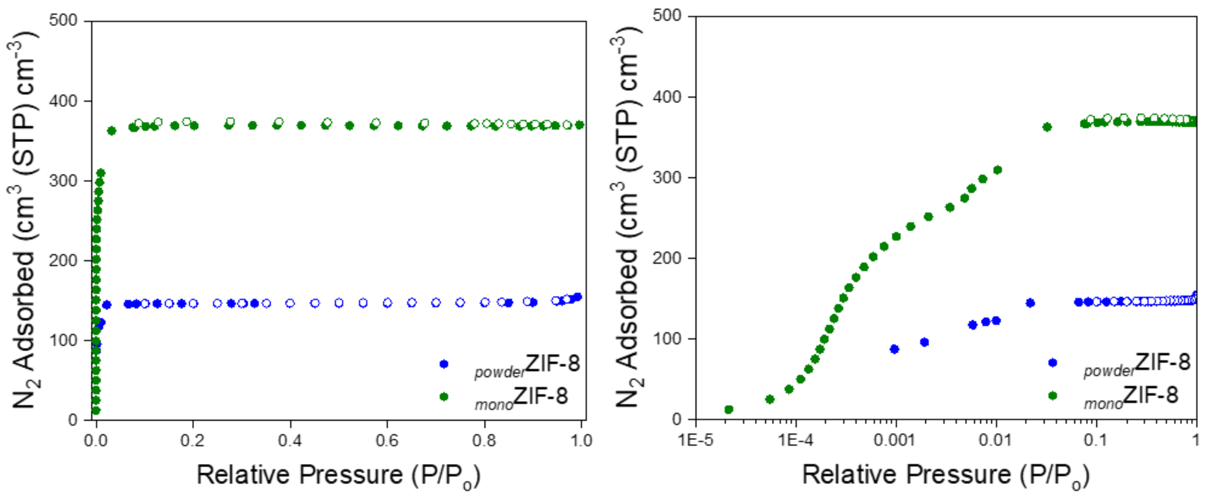

Figure S3. Volumetric 77 K N_2 isotherms for $_{powder}$ZIF-8 and $_{mono}$ZIF-8 in (Left) linear and (Right) logarithmic scale. Closed symbols represent adsorption, open symbols represent desorption.

3. Brunauer–Emmett–Teller (BET) area calculation using BET Surface Identification (BETSI)

BETSI is based on the original Rouquerol criteria for reporting BET areas, but is modified to prevent manual interaction. The only input data required is the adsorption isotherm.

The Rouqurol criteria are as follows:

Regression criteria:

- The linear range should span at least 10 points.
- The residual R^2 should be greater than or equal to 0.995.

Validity criteria:

1. Over the entire fitting range, $N(1 - \frac{P}{P_0})$ must increase monotonically with $\frac{P}{p_0}$.
2. The value of C obtained by linear regression must be positive.

Self-consistency criteria:

4. The monolayer loading, when reported on the isotherm, $N_m(Read)$, must correspond to a pressure that lies in the linear region.
5. The relative pressure corresponding to the monolayer loading as obtained from BET theory, $\frac{p}{p_0}\ (N_m\ BET)$ must be equal to the pressure determined in criterion 3 within a 20% tolerance.

A detailed explanation of the output obtained from BETSI is given in **Figure SI3.**

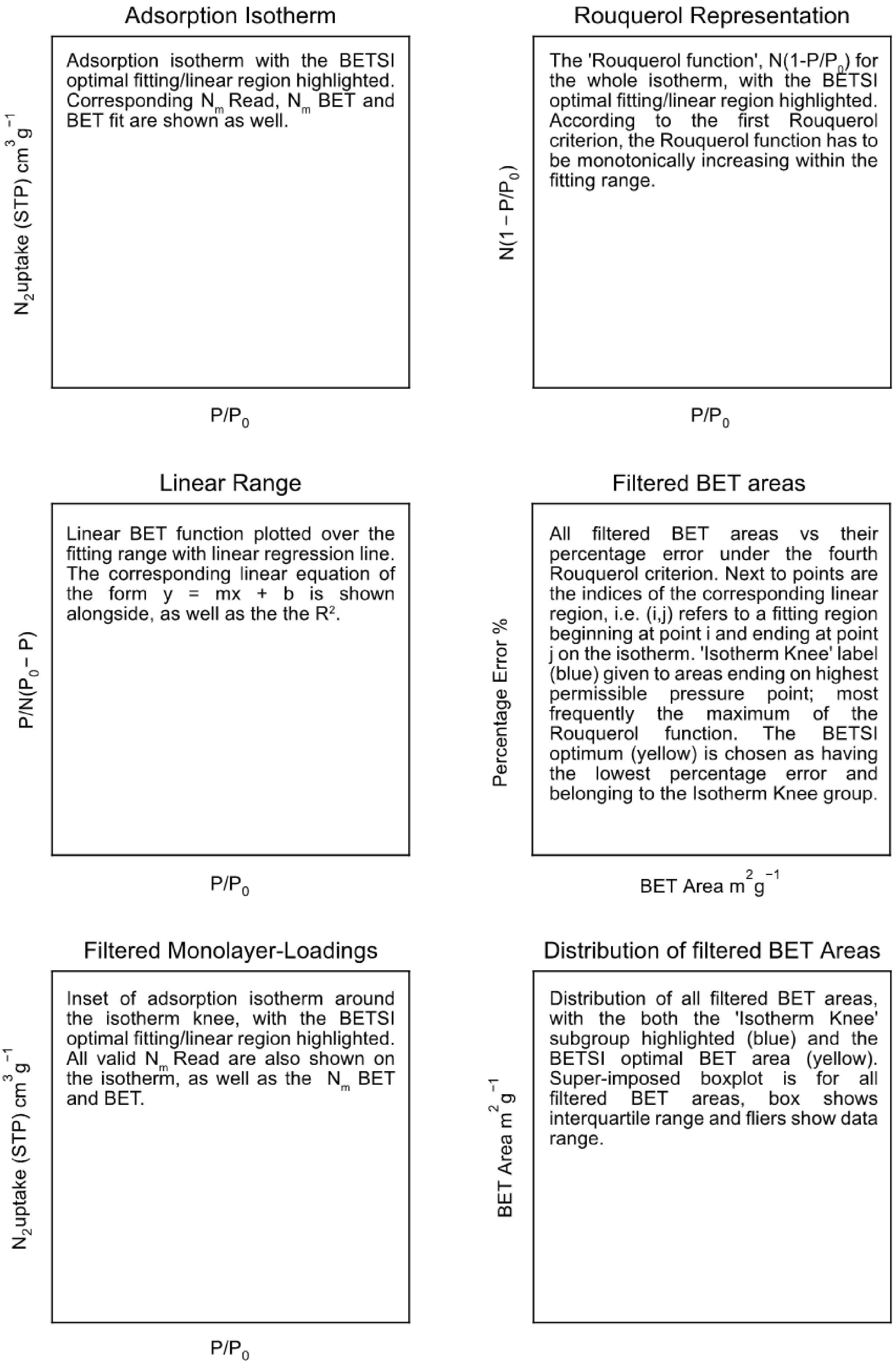

Figure S4 Description of the different plots in Figure SI4 obtained in a BETSI analysis

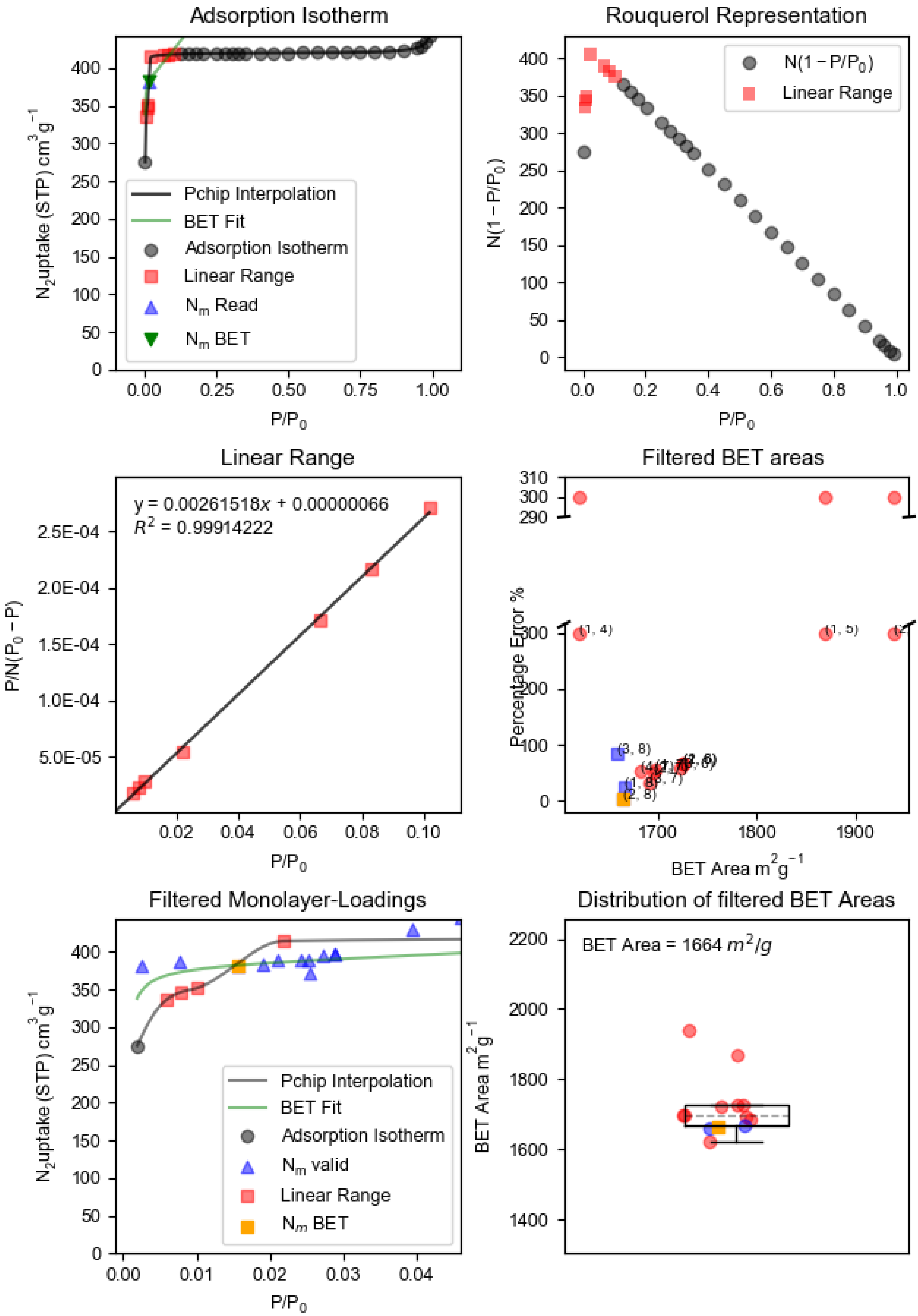

Figure S5. BETSI analysis of $_{powder}$ZIF-8

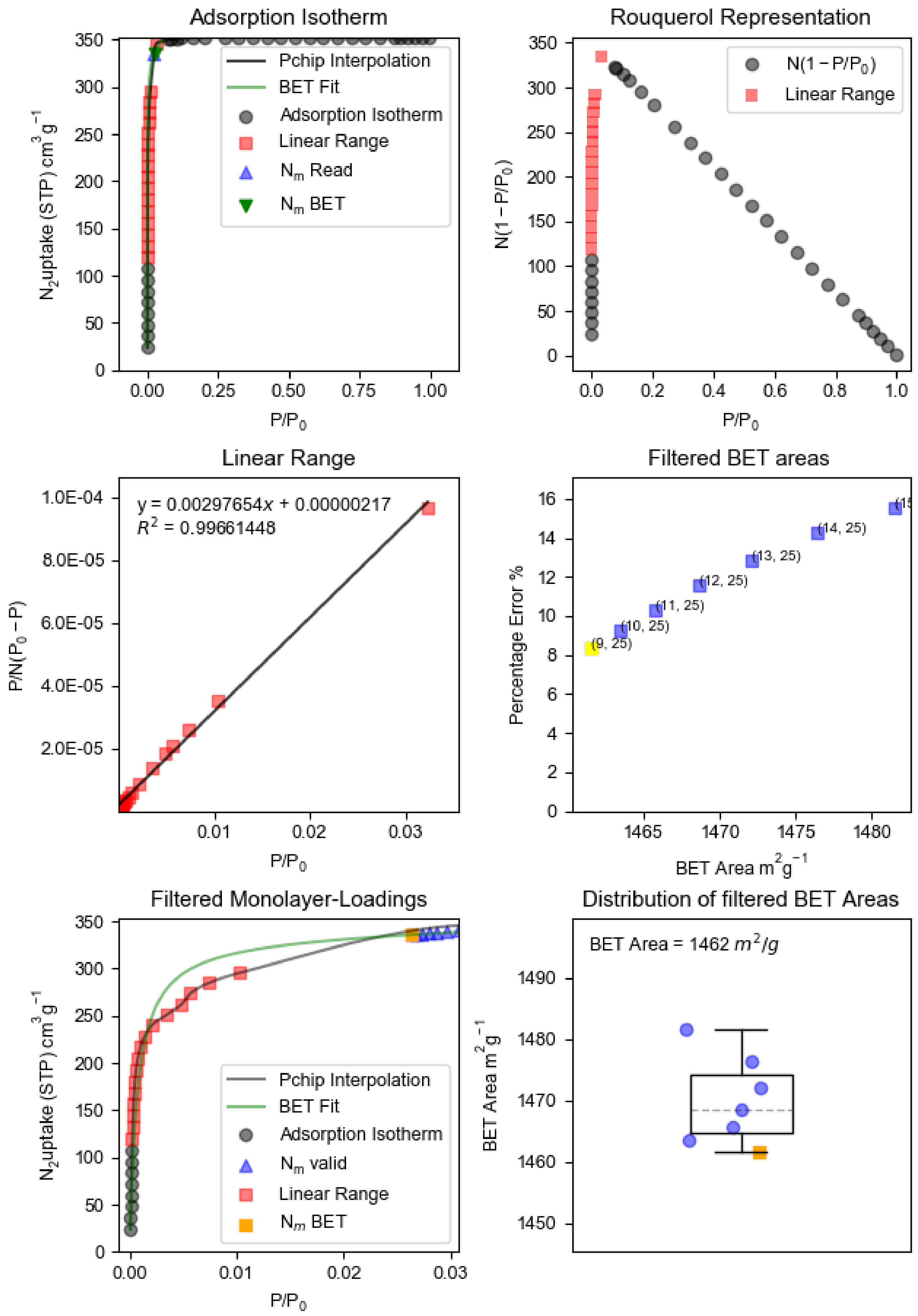

Figure S6. BETSI analysis of $_{mono}$ZIF-8

4. Intrusion-extrusion isotherms normalized per initial volume

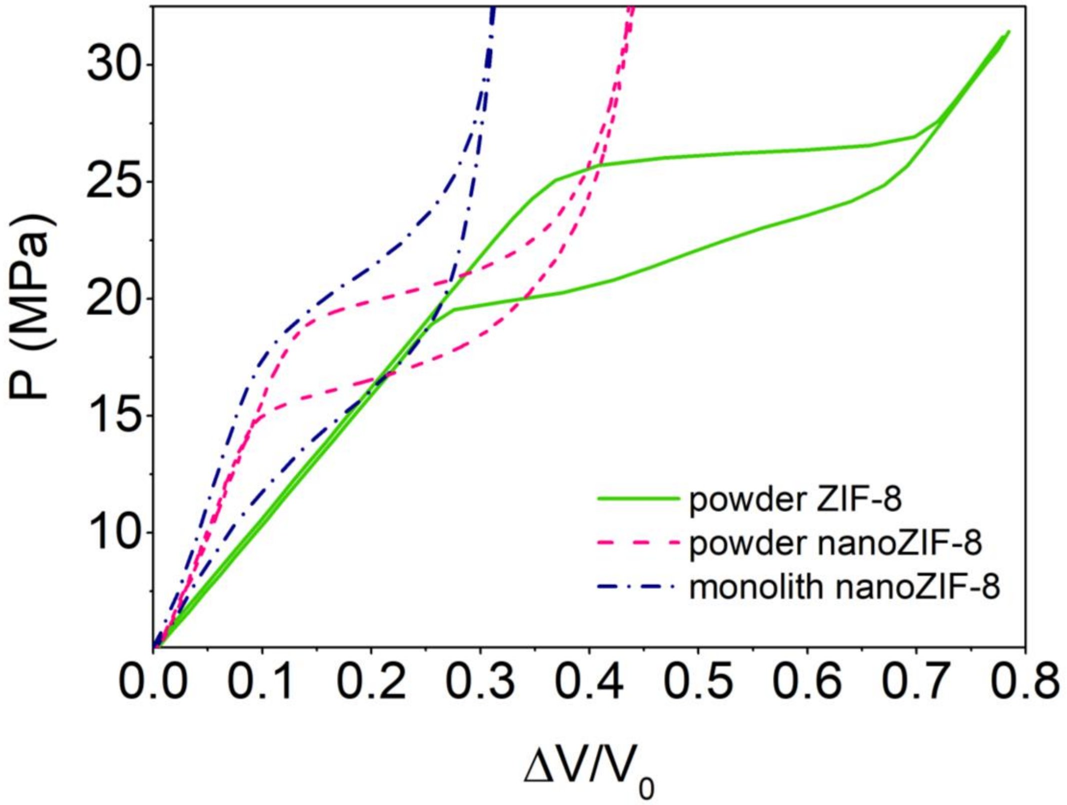

Figure S7. Room temperature *PV*-isotherms for ZIF-8 + water system: comparison between $_{powder}$ZIF-8, $_{powder_nano}$ZIF-8 and $_{mono_nano}$ZIF-8 at 0.1 MPa/min compression rate. V_0 is the initial volume of the sample.

Figure S7 was achieved by multiplying ΔV recorded during intrusion-extrusion cycling (Figure 3a) by crystallographic density for each sample, which was obtained from XRD analysis and is presented in Table S1.

Table S1. Crystallographic density, lattice volume and lattice parameter for different ZIF-8 samples

	$_{powder}$ZIF-8	$_{powder_nano}$ZIF-8 and	$_{mono_nano}$ZIF-8
**Lattice parameter *a*, Å**	17.030(2)	16.975(3)	16.977(4)
**Lattice volume, Å^3**	4938.7 ± 0.8	4891.2 ± 1.5	4892.8 ± 1.5
**Density, g/cm^3**	0.91807	0.92702	0.92670

5. **Experiments related to intrusion volume differences**

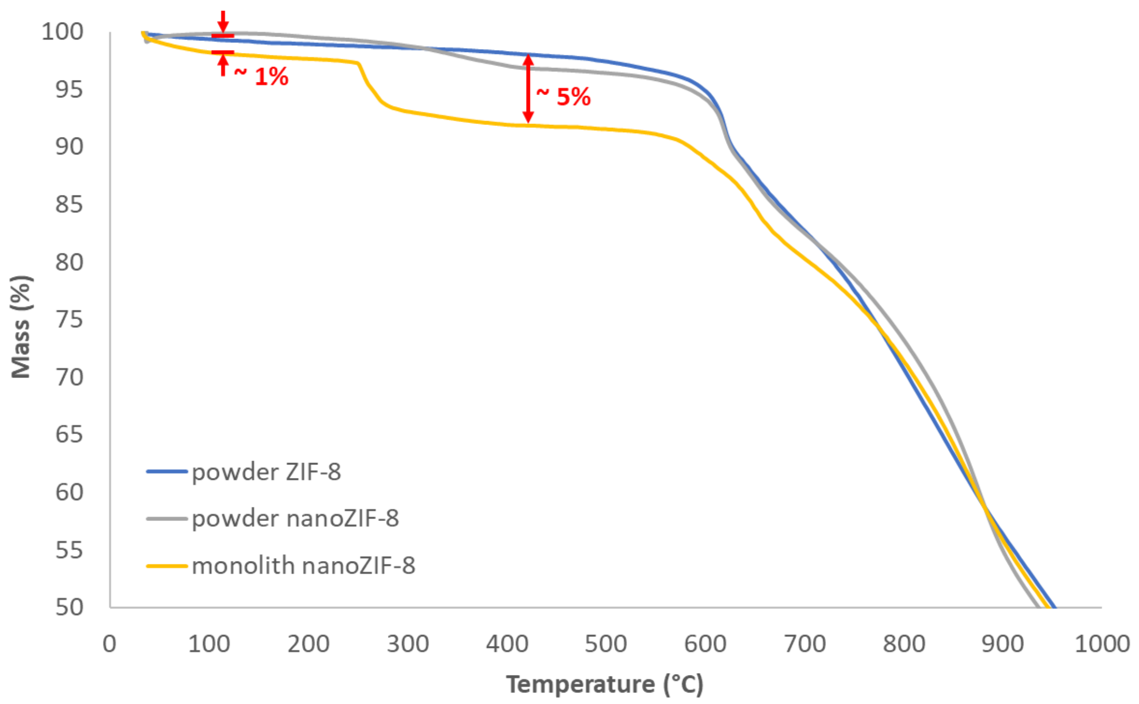

Figure S8. Thermogravimetric test for ZIF-8 samples after maintaining them under 90% humidity for 24 hours

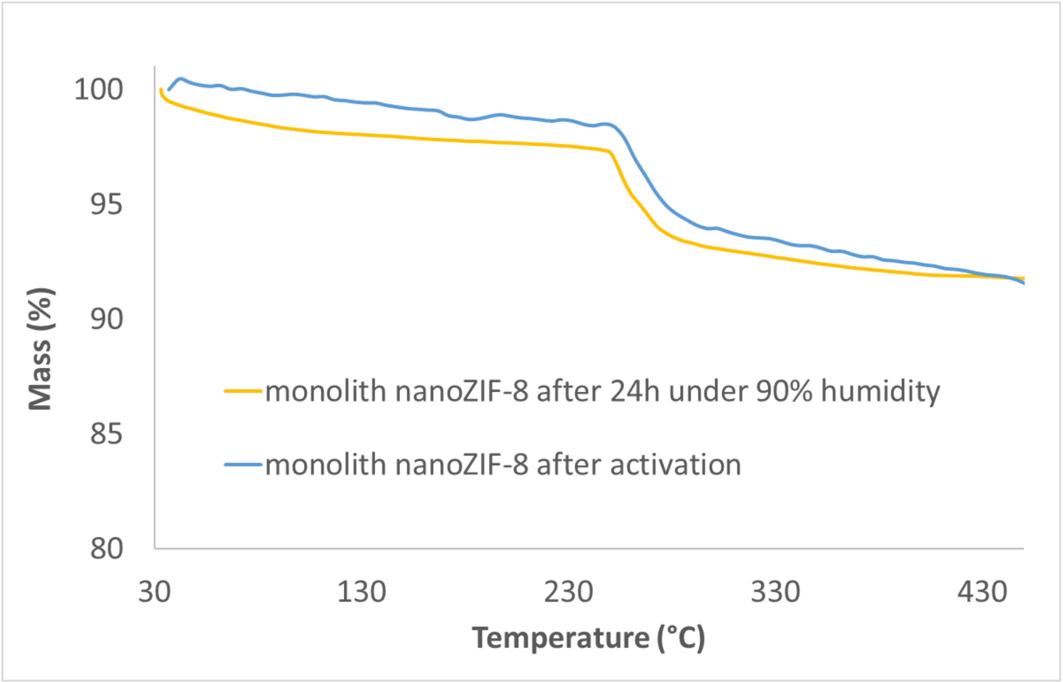

Figure S9. Thermogravimetric test for $_{mono_nano}$ZIF-8 sample after maintaining it under 90% humidity for 24 hours and after activation under vacuum at 100°C

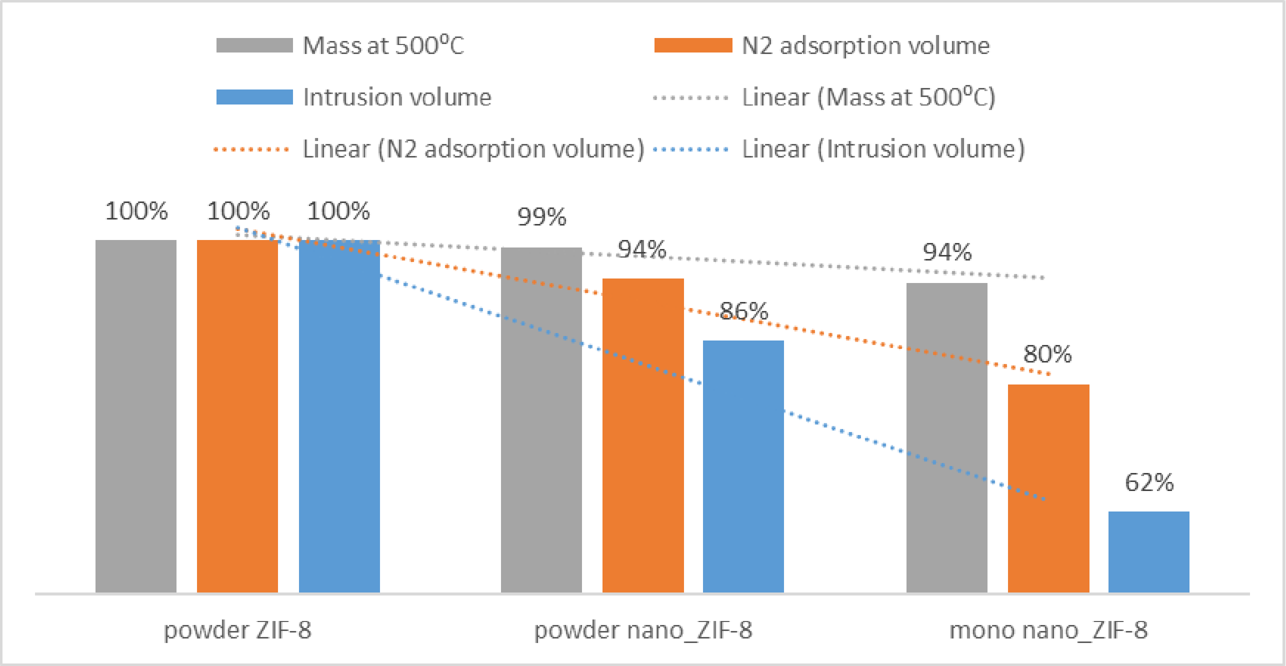

Figure S10. Relative intrusion volume, N_2 adsorption volume and mass at 500°C for different ZIF-8 samples. The values for powder ZIF-8 are taken as 100%. The values for powder nanoZIF-8 and mono nanoZIF-8 are calculated as percentage of the corresponding values of powder ZIF-8

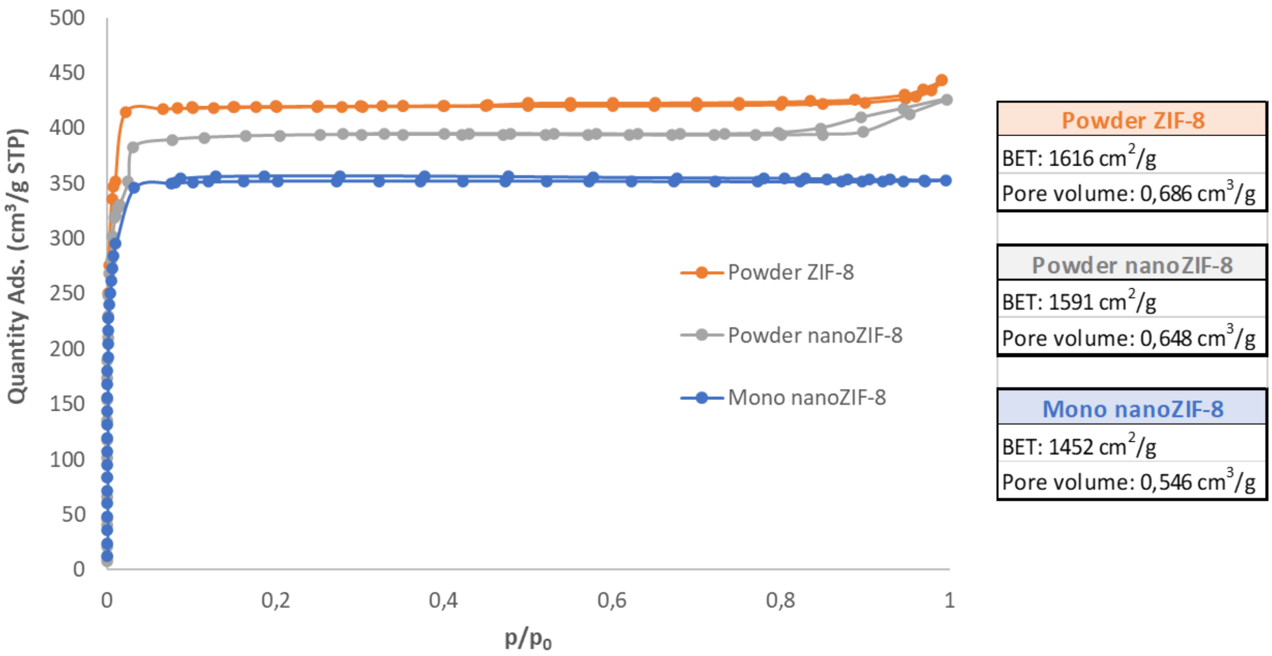

Figure S11. N_2 adsorption isotherms for different ZIF-8 samples

6. Stability of monolith samples

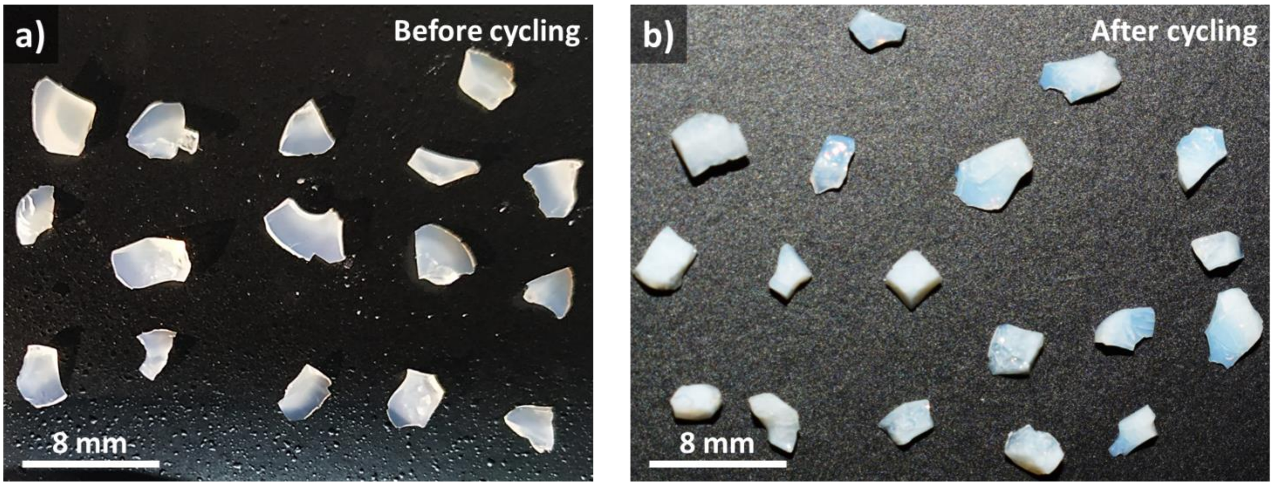

Figure S12. Photographs of monolith nanoZIF-8 before (a) and after (b) intrusion-extrusion cycling

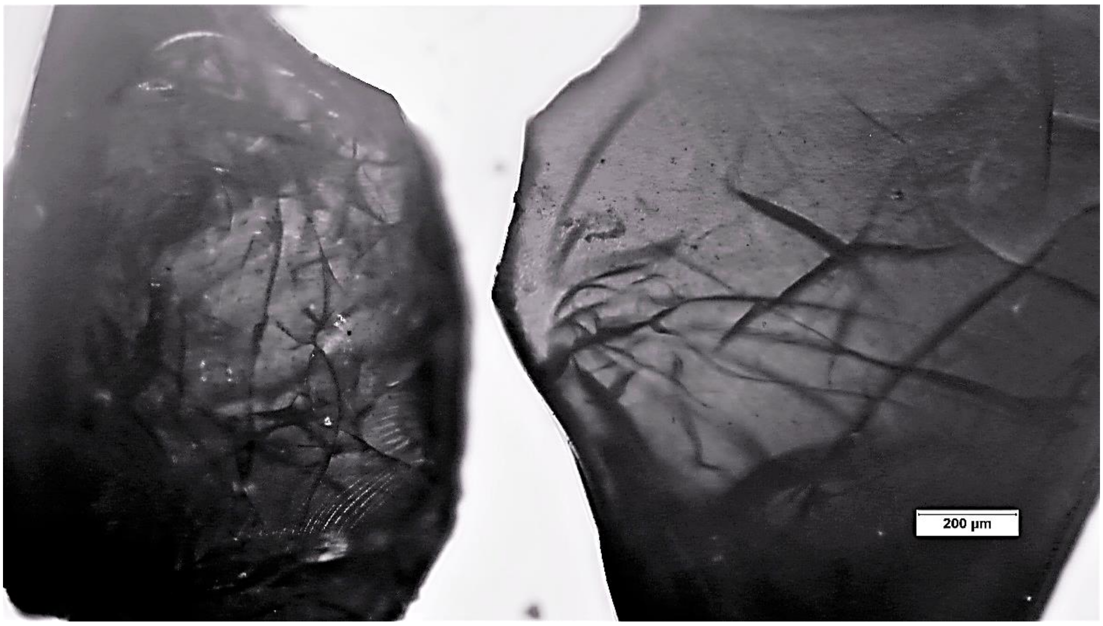

Figure S13. Optical image of monolith nanoZIF-8 after intrusion-extrusion cycling. Formation of some cracks can be seen.

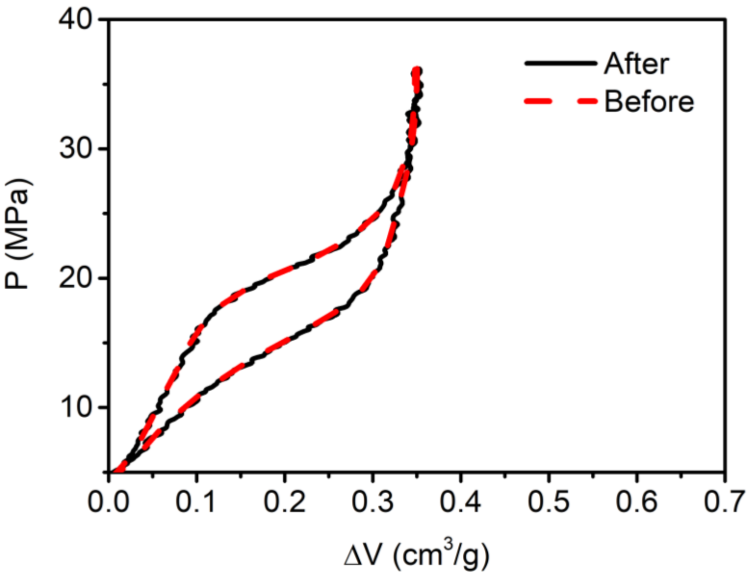

Figure S14. Intrusion-extrusion cycle of monolith for nanoZIF-8 before and after intrusion-extrusion cycling

7. String method

Intrusion and percolation paths of a water molecule across the 6-member ring windows and along the grain boundary (GB) have been determined using the string method.[1,2] This method allows one to identify the most likely transition path, i.e., the optimal path connecting initial and final states among the many. Along this path, we determined the energetics of the process, i.e., the relative energy of the initial and final state, and the transition barrier, the barrier the water molecule has to overcome to accomplish the process.

In the string method, one represents the transition path as a parametric curve, $\mathbf{r}(\lambda)$, in the space of the atomic configurations ($\mathbf{r}$ is the 3N vector of the atomic positions). λ is a parameter measuring the degree of progress of the transition: λ = 0 when the systems are in the initial state, and λ = 1 when it is in the final one. It can be shown[1,2] that the most probable path is the one with zero component of the atomic forces in the direction orthogonal to the path:

$$[-\nabla V(\mathbf{r}(\lambda))]_{\perp\mathbf{r}(\lambda)} = 0$$

In the string method, the continuous path (λ) is discretized into a finite number, $\boldsymbol{L}$, of configurations (snapshots), $\{\mathbf{r}(\lambda_i)\}_{\boldsymbol{i=1,L}}$. These snapshots satisfy the additional condition to be at a constant distance from each other: $|\mathbf{r}(\lambda_i) - \mathbf{r}(\lambda_{i+1})| = |\mathbf{r}(\lambda_j) - \mathbf{r}(\lambda_{j+1})|$.

Starting from an initial guess, the path is evolved according to the steepest descent dynamics driven by the component of the forces perpendicular to the path: $-\nabla V(\mathbf{r}(\lambda_i))(\mathbf{1} - \alpha(\mathbf{r}(\lambda_i))\otimes\alpha(\mathbf{r}(\lambda_i)))$, whit $\mathbf{1}$ identity matrix and $\alpha(\mathbf{r}(\lambda_i))$ unit vector tangent to the path at the i-th point; in practice, $(\mathbf{1} - \alpha(\mathbf{r}(\lambda_i))\otimes\alpha(\mathbf{r}(\lambda_i)))$ is a projector on the plane orthogonal to the path at the point $\mathbf{r}(\lambda_i)$. Indeed, since the tangential component of the force has only the effect of changing the distance between successive *snapshots*, a more efficient algorithm consists in i) evolving the path according to the entire force and ii) imposing the equidistance condition by *moving* the snapshots along the polygonal joining the configurations $\{\mathbf{r}(\lambda_i)\}_{\boldsymbol{i=1,L}}$ obtained in i). This two-step process is repeated until the maximum component of the atomistic force orthogonal to the path is below 10^{-3} Hartree/Bohr (atomic units), the same convergence criterion that is typically used in cell optimizations. On the converged string we apply the "climbing algorithm", which moves the higher in energy snapshot upward, toward the maximum of the energy along the path. This allows one to accurately determine the transition barrier.

String calculations were performed using the neb.x module of the Quantum Espresso package.[3]

8. Free Energy Calculations

In this work, we use restrained molecular dynamics, RMD,[4,5] to compute the (Landau) free energy profile and other properties of the system as a function of the level of filling of the ZIF-8 slab by water. In statistical mechanics, any thermodynamic potential is related to the logarithm of a suitable probability density function in the relevant ensemble. In the present case, the relevant probability density function is $M\left(N^*_{H_2O}\right)$, the probability density that the ZIF-8 crystallite contains a given number of H_2O molecules. In terms of the ensemble distribution $m(\boldsymbol{r})$, i.e., given the probability density to find the atoms of the system in the position corresponding to the $3N$ dimensional vector $\boldsymbol{r}$:

$$M\left(N_{H_2O}^{*}\right) = \int \boldsymbol{dr}\ m(\boldsymbol{r})\ \delta\left(\widehat{N}_{H_2O}(\boldsymbol{r}) - N_{H_2O}^{*}\right) \tag{1}$$

where $\delta(\cdot)$ is the Dirac delta function, which has the role of selecting only those atomic configurations satisfying the condition that the present number of water molecules in the slab, $\widehat{N}_{H_2O}(\boldsymbol{r})$, is equal to the target value, $N_{H_2O}^{*}$.

One can associate a thermodynamic potential, the Landau free energy, to $M\left(N_{H_2O}^{*}\right)$ (see, e.g., Refs. [2,6]).

$$G\left(N_{H_2O}^{*}\right) = -k_B T \log M\left(N_{H_2O}^{*}\right) \tag{2}$$

where $k_B T$ is the thermal energy at the experimental temperature T (k_B is the Boltzmann constant).

To compute $M\left(N_{H_2O}^{*}\right)$ and, therefore $G\left(N_{H_2O}^{*}\right)$ from atomistic simulations one can in principle run a long molecular dynamics trajectory and determine the histogram of $N_{H_2O}^{*}$ along it: this histogram is a discrete approximation of $M\left(N_{H_2O}^{*}\right)$, from which one can compute the free energy *via* Eq. (2). However, it is well known that this approach is computationally inefficient (see, e.g., Ref.[2,6,7] and references cited therein). This is because in presence of large free energy barriers, larger than the thermal energy $k_B T$, within the typical duration of a simulation (from tens of nanoseconds to maximum microseconds) the system visits only the basis around the initial configuration.

To overcome this problem, we use RMD[4,5] (see also Refs.[8,9]), in which one introduces a controlled bias forcing the system to explore configurations corresponding to a given number of molecules in the ZIF-8 slab. Consider the (Landau) free energy of Eq. (2) and, for the sake of simplicity, assume that the ensemble is canonical, hence $m(\boldsymbol{r}) = \exp\left[-V(\boldsymbol{r})/k_B T\right] / \int \boldsymbol{dr} \exp\left[-V(\boldsymbol{r})/k_B T\right]$, where $V(\boldsymbol{r})$ is the (physical) interacting potential, the so-called *force field*. We remark that the arguments developed below can be straightforwardly extended to other ensembles, e.g., isothermal-isobaric. Within the canonical ensemble the probability density function of Eq. (1) reads:

$$M\left(N_{H_2O}^{*}\right) = \frac{\int \boldsymbol{dr} \exp\left[-V(\boldsymbol{r})/k_B T\right] \delta\left(\widehat{N}_{H_2O}(\boldsymbol{r}) - N_{H_2O}^{*}\right)}{\int \boldsymbol{dr} \exp\left[-V(\boldsymbol{r})/k_B T\right]} \tag{4}$$

Hence, the derivative of the free energy is

$$\frac{dG\left(N_{H_2O}^{*}\right)}{dN_{H_2O}^{*}} = -k_B T \frac{\int \boldsymbol{dr} \exp\left[-V(\boldsymbol{r})/k_B T\right] \, d\left[\delta\left(\widehat{N}_{H_2O}(\boldsymbol{r}) - N_{H_2O}^{*}\right)\right] \Big/ dN_{H_2O}^{*}}{\int \boldsymbol{dr} \exp\left[-V(\boldsymbol{r})/k_B T\right] \delta\left(\widehat{N}_{H_2O}(\boldsymbol{r}) - N_{H_2O}^{*}\right)} \tag{5}$$

$G\left(N_{H_2O}^{*}\right)$ can be computed by numerical integration of $dG\left(N_{H_2O}^{*}\right)/dN_{H_2O}^{*}$. The advantage is that, as we will show shortly, the derivative of the free energy can be more easily estimated by atomistic simulations. To achieve this objective, in Eq. (5) one replaces the Dirac delta functions with a smooth Gaussian approximation,

$$\delta\left(\widehat{N}_{H_2O}(\boldsymbol{r}) - N_{H_2O}^{*}\right) \sim g_\lambda\left(\widehat{N}_{H_2O}(\boldsymbol{r}) - N_{H_2O}^{*}\right) = \sqrt{2\pi k_B T/\lambda}\ \exp\left[-\frac{k}{2}\left(\widehat{N}_{H_2O}(\boldsymbol{r}) - N_{H_2O}^{*}\right)^2 / k_B T\right] \tag{6}$$

Here, $k_B T/k$ is the standard deviation of the Gaussian function, the parameter determining its width and, thus, the accuracy of the approximation to the corresponding Dirac delta function. The reason to express the standard deviation of the gaussian by the apparently more complex form $k_B T/k$ will be clear shortly. Within the Gaussian approximation the derivative of the free energy, Eq (5), reads:

$$\frac{dG\left(N^*_{H_2O}\right)}{dN^*_{H_2O}} \sim \frac{\int d\boldsymbol{r}\, k\left(\widehat{N}_{H_2O}(\boldsymbol{r})-N^*_{H_2O}\right)\, \exp\left\{-\left[V(\boldsymbol{r})+\frac{k}{2}\left(\widehat{N}_{H_2O}(\boldsymbol{r})-N^*_{H_2O}\right)^2\right]/k_BT\right\}}{\int d\boldsymbol{r}\, \exp\left\{-\left[V(\boldsymbol{r})+\frac{k}{2}\left(\widehat{N}_{H_2O}(\boldsymbol{r})-N^*_{H_2O}\right)^2\right]/k_BT\right\}} \quad (7)$$

Thus, within the Gaussian approximation of the Dirac delta function, the derivative of the free energy can be computed as the expectation value of $k\left(\widehat{N}_{H_2O}(\boldsymbol{r}) - N^*_{H_2O}\right)$ over the canonical ensemble of a system driven by the so-called augmented potential $V(\boldsymbol{r}) + \frac{k}{2}\left(\widehat{N}_{H_2O}(\boldsymbol{r}) - N^*_{H_2O}\right)^2$. In practice, one computes $dG\left(N^*_{H_2O}\right)/dN^*_{H_2O}$ at the current value of $N^*_{H_2O}$ by averaging the observable $k\left(\widehat{N}_{H_2O}(\boldsymbol{r}) - N^*_{H_2O}\right)$ along the trajectory of a constant number of particles/volume/temperature molecular dynamics driven by the potential $V(\boldsymbol{r}) + \frac{k}{2}\left(\widehat{N}_{H_2O}(\boldsymbol{r}) - N^*_{H_2O}\right)^2$. The operation is repeated for several values of $N^*_{H_2O}$ in the interval 0, ZIF-8 empty of water, and 1500, ZIF-8 full of water; then, the so-obtained $dG\left(N^*_{H_2O}\right)/dN^*_{H_2O}$ is numerically integrated by the trapezoid rule.

Error on the free energy

The error on a derived observable $O = O(s)$, with s the variable that is directly measured, is usually obtained by error propagation:

$$\delta O^2 = \left(\frac{dO(s)}{ds}\delta s\right)^2 \quad (8)$$

Here, δs^2 and δO^2 are the variances of s and the estimated variance of O, respectively. Assuming that the error on O is a pure statistical error, δO or low multiples - $2 \times \delta O$, $3 \times \delta O$ – are a good estimation of error. However, δO obtained from error propagation is an upper bound of the actual statistical error of a derived observable.

In free energy calculations thermodynamic integration or analogous techniques, e.g., RMD, which the free energy G is obtained by numerical integration of $dG\left(N^*_{H_2O}\right)/dN^*_{H_2O}$, Eq. 7, error propagation, Eq. 8, brings to a severe overestimation of the error on $G\left(N^*_{H_2O}\right)$:

$$\delta G\left(N_j\right)^2 = \sum_{i=1,j} \frac{G'(N_i)^2 + G'(N_{i-1})^2}{2}\left(N_i - N_{i-1}\right)^2 \quad (9)$$

Where $G'(\cdot)$ is just a more compact notation of $dG\left(N^*_{H_2O}\right)/dN^*_{H_2O}$, N_i represents the number of water molecules in the ZIF-8 slab, which in our RMD calculation changes in steps of 10.

Here, like in previous works,[8,10–14] we use a different approach. We divide the configurations used to estimate $dG\left(N^*_{H_2O}\right)/dN^*_{H_2O}$ in M smaller sets from which we obtain the corresponding estimates of the mean force $\left\{\left(dG\left(N^*_{H_2O}\right)/dN^*_{H_2O}\right)_i\right\}_{i=1,M}$. For each series of the gradient of free energy, for all values of $N^*_{H_2O}$, by numerical integration, one obtains a set of free energy curves $\left\{\left(G\left(N^*_{H_2O}\right)\right)_i\right\}_{i=1,M}$, which can be used to directly compute the variance $\delta G\left(N_j\right)^2$ at each value N_j of water molecules in the ZIF-8 slab:

$$\delta G\left(N_j\right)^2 = \frac{1}{M-1}\sum_{i=1,M}\left[\left(G\left(N_j\right)\right)_i - G\left(N_j\right)\right]^2 \quad (10)$$

Here, $G(N_j)$ is obtained from the numerical integration of $dG(N^*_{H_2O})/dN^*_{H_2O}$ determined with the complete set of simulation data. Correlation effects can be properly taken into account by using standard techniques such as, for example, the *block average* or the *Jackknife* methods.[15] The average error on the free energy (Fig. SI3), after data correlation has been taken into account, is 5k_BT.

9. References

(1) Grimme, S. Accurate Description of van Der Waals Complexes by Density Functional Theory Including Empirical Corrections. *J. Comput. Chem.* **2004**, *25* (12), 1463–1473. https://doi.org/10.1002/jcc.20078.

(2) Bonella, S.; Meloni, S.; Ciccotti, G. Theory and Methods for Rare Events. *Eur. Phys. J. B 2012 853* **2012**, *85* (3), 1–19. https://doi.org/10.1140/EPJB/E2012-20366-2.

(3) Giannozzi, P.; Baroni, S.; Bonini, N.; Calandra, M.; Car, R.; Cavazzoni, C.; Ceresoli, D.; Chiarotti, G. L.; Cococcioni, M.; Dabo, I.; Dal Corso, A.; De Gironcoli, S.; Fabris, S.; Fratesi, G.; Gebauer, R.; Gerstmann, U.; Gougoussis, C.; Kokalj, A.; Lazzeri, M.; Martin-Samos, L.; Marzari, N.; Mauri, F.; Mazzarello, R.; Paolini, S.; Pasquarello, A.; Paulatto, L.; Sbraccia, C.; Scandolo, S.; Sclauzero, G.; Seitsonen, A. P.; Smogunov, A.; Umari, P.; Wentzcovitch, R. M. QUANTUM ESPRESSO: A Modular and Open-Source Software Project for Quantum Simulations of Materials. *J. Phys. Condens. Matter* **2009**, *21* (39). https://doi.org/10.1088/0953-8984/21/39/395502.

(4) Maragliano, L.; Vanden-Eijnden, E. A Temperature Accelerated Method for Sampling Free Energy and Determining Reaction Pathways in Rare Events Simulations. *Chem. Phys. Lett.* **2006**, *426* (1–3), 168–175. https://doi.org/10.1016/J.CPLETT.2006.05.062.

(5) Abrams, J. B.; Tuckerman, M. E. Efficient and Direct Generation of Multidimensional Free Energy Surfaces via Adiabatic Dynamics without Coordinate Transformations. *J. Phys. Chem. B* **2008**, *112* (49). https://doi.org/10.1021/jp805039u.

(6) Peters, B. *Reaction Rate Theory and Rare Events*; 2017.

(7) Giacomello, A.; Chinappi, M.; Meloni, S.; Casciola, C. M. Metastable Wetting on Superhydrophobic Surfaces: Continuum and Atomistic Views of the Cassie-Baxter–Wenzel Transition. *Phys. Rev. Lett.* **2012**, *109* (22), 226102. https://doi.org/10.1103/PhysRevLett.109.226102.

(8) Giacomello, A.; Meloni, S.; Chinappi, M.; Casciola, C. M. Cassie–Baxter and Wenzel States on a Nanostructured Surface: Phase Diagram, Metastabilities, and Transition Mechanism by Atomistic Free Energy Calculations. *Langmuir* **2012**, *28* (29), 10764–10772. https://doi.org/10.1021/LA3018453.

(9) Meloni, S.; Ciccotti, G. Free Energies for Rare Events: Temperature Accelerated MD and MC. *European Physical Journal: Special Topics*. 2015. https://doi.org/10.1140/epjst/e2015-02418-7.

(10) Amabili, M.; Giacomello, A.; Meloni, S.; Casciola, C. M. Intrusion and Extrusion of a Liquid on Nanostructured Surfaces. *J. Phys. Condens. Matter* **2016**, *29* (1), 014003. https://doi.org/10.1088/0953-8984/29/1/014003.

(11) Amabili, M.; Giacomello, A.; Meloni, S.; Casciola, C. M. Unraveling the Salvinia Paradox: Design

Principles for Submerged Superhydrophobicity. *Adv. Mater. Interfaces* **2015**, *2* (14), 1500248. https://doi.org/10.1002/ADMI.201500248.
(12) Amabili, M.; Meloni, S.; Giacomello, A.; Casciola, C. M. Activated Wetting of Nanostructured Surfaces: Reaction Coordinates, Finite Size Effects, and Simulation Pitfalls. *J. Phys. Chem. B* **2017**, *122* (1), 200–212. https://doi.org/10.1021/ACS.JPCB.7B07429.
(13) Amabili, M.; Giacomello, A.; Meloni, S.; Casciola, C. M. Collapse of Superhydrophobicity on Nanopillared Surfaces. *Phys. Rev. Fluids* **2017**, *2* (3), 034202. https://doi.org/10.1103/PhysRevFluids.2.034202.
(14) Lisi, E.; Amabili, M.; Meloni, S.; Giacomello, A.; Casciola, C. M. Self-Recovery Superhydrophobic Surfaces: Modular Design. *ACS Nano* **2017**, *12* (1), 359–367. https://doi.org/10.1021/ACSNANO.7B06438.
(15) Janke, W. Statistical Analysis of Simulations: Data Correlations and Error Estimation. *Quantum* **2002**, *10*.
